# Biocatalytic Nanoregulators Restore Joint Redox‐Immune Homeostasis in Rheumatoid Arthritis

**DOI:** 10.1002/advs.202502894

**Published:** 2026-01-14

**Authors:** Xingheng Wang, Jianbo Huang, Shuwei Zhang, Sujiao Cao, Fangxue Du, Liqiang Zhou, Li Qiu, Yuanjiao Tang

**Affiliations:** ^1^ Department of Ultrasound National Clinical Research Center for Geriatrics Med‐X Center for Materials West China Hospital Sichuan University Chengdu 610041 China; ^2^ MOE Frontiers Science Center for Precision Oncology Faculty of Health Sciences University of Macau Macau SAR 999078 China

**Keywords:** anti‐inflammatory, extracellular vesicles, joint protection, macrophage polarization, rheumatoid arthritis, targeted therapy

## Abstract

Rheumatoid arthritis (RA), a debilitating autoimmune disorder marked by progressive synovitis and osteochondral erosion, presents critical therapeutic challenges due to the limitations of existing regimens, including non‐specific biodistribution and compromised risk‐benefit profiles. In this study, a novel therapeutic approach is proposed utilizing mesenchymal stem cell‐derived extracellular vesicles (EVs) coating on ruthenium‐loaded metal‐organic frameworks (Ru@ZrMOF), which exhibit catalase mimetic activities. The EVs coating enhances the biocompatibility and targeting efficiency of the Ru@ZrMOF, while also promoting cartilage protection. Through reactive oxygen species (ROS) scavenging and oxygen production, Ru@ZrMOF/EVs significantly alleviate joint inflammation, promote cartilage protection, and inhibit pannus formation. The therapeutic mechanism involves polarization of macrophages from the M1 pro‐inflammatory phenotype to the M2 anti‐inflammatory phenotype, facilitating a shift in cytokine profiles from pro‐inflammatory to anti‐inflammatory, and downregulation of hypoxia inducible factor‐1α. These findings demonstrate that Ru@ZrMOF/EVs offer a promising strategy for RA treatment by addressing both inflammation and tissue protection through targeted ROS regulation and immune modulation.

## Graphical abstract

Mesenchymal stem cell‐derived extracellular vesicles (EVs) coat ruthenium‐loaded metal‐organic frameworks (Ru@ZrMOF), creating a targeted therapeutic (Ru@ZrMOF/EVs). This platform scavenges ROS, generates oxygen, and polarizes macrophages from M1 to M2, alleviating inflammation, inhibiting pannus, promoting cartilage repair, and downregulating HIF‐1α in RA joints.

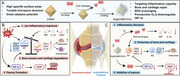

## 1 Introduction

Rheumatoid arthritis (RA) is a chronic systemic disease mainly manifested by multi‐joint inflammation, which can eventually lead to joint destruction and deformity.^[^ ^1^, ^2^, ^3^ ^]^ RA has a high global prevalence of 0.5–1.0%.^[^ ^4^ ^]^ Current clinical RA treatment agents include non‐steroidal anti‐inflammatory drugs, disease‐modifying antirheumatic drugs, and biological agents for antagonizing inflammatory cytokines such as tumor necrosis factor‐α (TNF‐α) inhibitors, while there can be long‐term drug dependence, considerable side effects, and poor efficacy in above 30–50% of patients.^[^ ^5^, ^6^ ^]^ However, existing therapeutic drugs cannot achieve cartilage protection. Therefore, promoting cartilage protection in RA has always been a clinical challenge, and exploring new methods to achieve cartilage protection is necessary. The joint synovial thickness in RA patients shows a remarkable increase caused by infiltrating fibroblast‐like synoviocytes and macrophages, which exacerbate synovial hyperplasia and foster inflammation and tissue damage by releasing proinflammatory cytokines like TNF‐α, interleukin‐1β (IL‐1β), interleukin‐6 (IL‐6).^[^ ^7^, ^8^, ^9^ ^]^The infiltration of inflammatory cells in the joint, and increased local oxygen demand lead to anoxia of the synovial tissue of the joint, resulting in upregulation of hypoxia inducible factor‐1α (HIF‐1α), which can lead to increased angiogenesis by upregulation vascular endothelial growth factor (VEGF), accelerating the RA formation of pannus.^[^ ^10^ ^]^ Meanwhile, the proinflammatory M1 phenotype of macrophages in inflamed synovial tissue can promote the expression of various proinflammatory factors, such as TNF‐α, IL‐6, hypoxia inducible factor 1α (HIF‐1α), which is also positively correlated with the generation of reactive oxygen species(ROS).^[^ ^11^, ^12^ ^]^ Thus, scavenging ROS, alleviating hypoxia, reducing neovascularization, and reprogramming macrophages can be an effective strategy for RA treatment.^[^ ^13^, ^14^, ^15^ ^]^

The catalytic scavenging of ROS by natural enzymes is a key biochemical reaction that maintains redox balance in tissue development and protection.^[^ ^16^ ^]^ In the natural antioxidase systems, catalase (CAT) can efficiently eliminate ROS by transforming hydrogen peroxide (H_2_O_2_) into H_2_O and O_2_.^[^ ^17^, ^18^ ^]^ However, the direct application of natural CAT in treating inflammatory diseases encounters several challenges, including antigenicity, poor stability post‐administration, and scalability issues, prompting increased focus on developing artificial enzyme‐mimetic catalytic activities.^[^ ^19^, ^20^ ^]^ We used metal–organic framework (MOF) with many intriguing traits including high specific surface area, tunable micropore structure by modulated synthesis, and great potential in catalysis.^[^ ^21^, ^22^ ^]^ Previous studies of our research group have shown that the ruthenium (Ru) complex has excellent antioxidase‐like activity.^[^ ^23^, ^24^, ^25^ ^]^ Thus, we doped Ru on the surface of ZrMOF synthesized to form Ru@ZrMOF, which has better CAT mimetic capacity and higher catalytic efficiency. Meanwhile, CAT mimetic nanomaterials have shown significant promise in treating RA by downregulating HIF‐1α expression and macrophages phenotype transformation from M1 to M2 recently.^[^ ^26^, ^27^, ^28^ ^]^ Promoting the polarization of macrophages from the M1 pro‐inflammatory phenotype to the M2 anti‐inflammatory phenotype, leading to a decrease in pro‐inflammatory markers such as iNOS, CD86, TNF‐α, and IL‐1β and an increase in anti‐inflammatory markers, including CD206, Arg‐1, and IL‐10.^[^ ^24^ ^]^ Additionally, mesenchymal stem cells‐derived extracellular vesicles (MSC‐EVs) could effective modulate pro‐inflammatory M1 to anti‐inflammatory M2 macrophages to mitigate the disease progression.^[^ ^29^ ^]^

MSC have been found to exert therapeutic effects in promoting the repair of bone and cartilage and tissue regeneration.^[^ ^30^, ^31^ ^]^ EVs are small‐sized (50–1000 nm) lipid bilayer membrane vesicles that are shed by the cell membrane or secreted by the cell, and are important mediators of intercellular communication.^[^ ^32^, ^33^ ^]^ MSC‐EVs mimic the cartilage repair effect of MSC by delivering various bioactive factors, which have been demonstrated to promote cartilage repair by inhibiting excessive chondrocyte death, promoting chondrocyte proliferation and differentiation, maintaining the chondrocyte phenotype, and regulating cartilage matrix metabolism.^[^ ^34^, ^35^, ^36^ ^]^ MSC‐EVs are advantageous in RA therapy due to outstanding features such as high biocompatibility, low immunogenicity, superior biological barrier penetration, and extended circulating half‐life.^[^ ^37^, ^38^ ^]^ Furthermore, MSC‐EVs can enhance drug delivery and increase retention at inflamed sites, making them promising candidates for targeted RA therapies.^[^ ^39^, ^40^ ^]^ Therefore, by coating natural EVs on Ru@ZrMOF can significantly enhance biocompatibility, targeting ability, and promote cartilage protection.

**Scheme 1:**
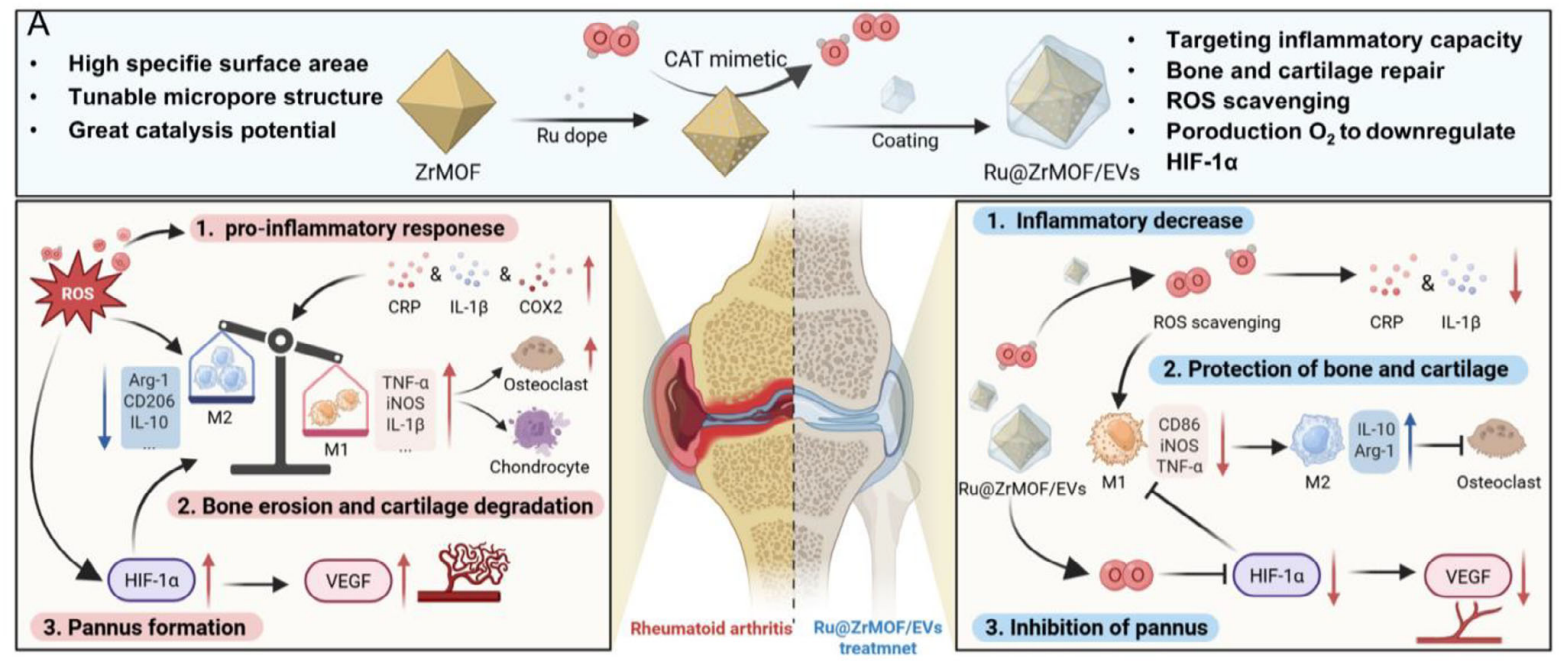
Illustrative construction and treatment mechanism of Ru@ZrMOF/EVs. A) Structural and biological application advantages of Ru@ZrMOF/EVs. B) Rheumatoid arthritis mechanism induced by ROS. C) Ru@ZrMOF/EVs treatingrheumatoid arthritis mechanism by reducing inflammatory, protection of bone and cartilage, and inhibition of pannus.

In this study, we propose a simple and novel design for EVs coating on Ru@ZrMOF (Ru@ZrMOF/EVs), which can promote cartilage protection and biocatalytic ROS scavenger function for efficiently suppressing the inflammatory in RA (**Scheme** **1**). We systematically investigate the benefits of Ru@ZrMOF/EVs, focusing on their targeted delivery to inflamed joints, ROS scavenging capabilities, macrophage polarization, and anti‐inflammatory effects. In vivo animal models of RA further corroborate the therapeutic efficacy of Ru@ZrMOF/EVs, showing that this treatment approach can alleviate RA joint inflammation, reduce bone erosion and cartilage degradation, and inhibit excessive neovascularization in inflamed joints. In summary, this study presents significant evidence showing that Ru@ZrMOF/EVs has contributed to inflammatory decrease, cartilage protection, and pannus inhibition, offering a promising approach for RA treatment.

## 2 Results and Discussion

### 2.1 Structural Characterization and ROS‐Scavenging Ability Evaluation

**Figure 1:**
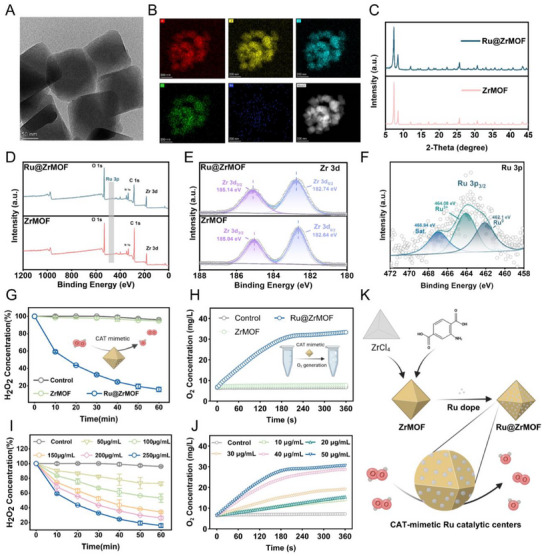
Structural characterization and ROS‐scavenging ability evaluation of Ru@ZrMOF. A) TEM of Ru@ZrMOF. B) EDS elemental mappings for C, N, O, Zr, and Ru elements and HAADF‐TEM in Ru@ZrMOF. C) XRD patterns of ZrMOF and Ru@ZrMOF. D) XPS survey spectra of ZrMOF and Ru@ZrMOF. E). The Zr 3d XPS analysis of ZrMOF and Ru@ZrMOF. F) The Ru 3p XPS analysis of Ru@ZrMOF. G) Time‐dependent CAT‐like performances in the presence of biocatalysts and H_2_O_2_ (*n* = 3 independent experiments, data are presented as mean ± S.D). H) Produced O_2_ concentration in the presence of biocatalysts and H_2_O_2_. I) Time‐dependent H_2_O_2_ decomposition activity of different concentrations of Ru@ZrMOF (*n* = 3 independent experiments, data are presented as mean ± S.D.). J) Produced O_2_ concentration at different concentrations of Ru@ZrMOF. K) Schematic of the construction of Ru@ZrMOF and the H_2_O_2_‐scavenging process.

We doped Ru particles on UiO‐66‐NH_2_, which is a universally used MOF with many intriguing traits including high specific surface area, tunable micropore structure by modulated synthesis, and great potential in catalysis. The Ru@ZrMOF biocatalyst was synthesized UIO‐66‐NH_2_ first. With low electronegativity and a higher metal state, Ru catalytic sites are enriched with electrons. This enrichment increases the occupancy of occupied orbitals in oxygen activation reactions and boosts their ability to exhibit antioxidase‐like activities. Thus, we chose Ru to construct UIO‐66‐NH_2_ with antioxidase‐like ROS‐scavenging ability. The Ru and UIO‐66‐NH_2_ were homodispersed in a RuCl_3_ aqueous solution to form the Ru‐doped ZrMOF. Scanning electron microscopy (SEM) images, transmission electron microscopy (TEM), and high angle annulardark field‐scanning transmission electron microscopy (HAADF‐TEM) images (Figures S1 and S2, Supporting Information; **Figure** **1**A) revealed both ZrMOF and Ru@ZrMOF present a nano octahedron morphology, verifying that the incorporation of Ru does not compromise the structure of ZrMOF. Similarly, Ru could be detected on the surface of Ru@ZrMOF. Energy‐dispersive spectroscopy (EDS) mapping of ZrMOF and Ru@ZrMOF, when compared, confirmed the successful incorporation of Ru in Ru@ZrMOF (Figures 1B; S2, Supporting Information). Additionally, EDS mapping revealed a uniform distribution of C, N, O, Zr, and Ru throughout the material. Nanoparticle tracking analysis (NTA) analysis revealed that the median hydrated diameter of the nanoparticles was 156.5 nm (Figure S3, Supporting Information). Figure 1C shows the X‐ray diffraction (XRD) patterns showed that the diffraction peak of Ru@ZrMOF was consistent with that of the ZrMOF. The primary framework architecture remained intact following the introduction of Ru ions. The composition and electronic structure of the Ru@ZrMOF nanoparticles were examined via X‐ray photoelectron spectroscopy (XPS). Figure 1D shows the XPS survey spectra in the presence of C, N, O, and Zr in the ZrMOF and C, N, O, Zr, and Ru in the Ru@ZrMOF. The high‐resolution Zr 3d spectra of the two nanomaterials could be deconvolved respectively into two peaks located at 182.74 and 185.14 eV, which were ascribed to Zr 3d_5/2_ and Zr 3d_3/2_ (Figure 1E). The Ru@ZrMOF, after being embedded with RuCl_3_, displayed a new peak at 281.8 eV, which can be assigned to Ru 3d_5/2_. As shown in Figure 1F, the high‐resolution Ru 3p spectrum was fitted by three peaks, and the peaks at 462.1, 464.08, and 466.94 eV derived from Ru^0^, Ru3^+^, a satellite peak.

As a crucial ROS agent, H_2_O_2_ provides the source for many other radical species in RA. Therefore, we examined the CAT‐mimicking properties of Ru@ZrMOF by monitoring the scavenging of H_2_O_2_ and the generation of O_2_. The Ru@ZrMOF shows a high H_2_O_2_ elimination rate with 53.0% decomposition of H_2_O_2_ in the first 30 min, whereas the ZrMOF could not eliminate H_2_O_2_ (Figure 1G). Meanwhile, the assessment of the generation of O_2_ showed that Ru@ZrMOF rapidly and thoroughly converted H_2_O_2_ into O_2_ and H_2_O (Figure 1H). In addition, Ru@ZrMOF exhibited a dose‐dependent response in H_2_O_2_ scavenging and O_2_ generation. At an extremely low concentration of 50 µg mL^−1^, Ru@ZrMOF achieved a 62.6% decomposition ratio in 60 min and an O_2_ generation rate of 30.29 mg L^−1^ in 3 min (Figure 1I,J). Hence, Ru doping enabled ZrMOF to exhibit efficient H_2_O_2_ scavenging activity (Figure 1K). To sum up, we successfully constructed the Ru@ZrMOF biocatalyst, which possesses significant CAT‐like activity for ROS scavenging applications.

### 2.2 Construction of Ru@ZrMOF/EVs and Targeting Capacity to Activated Macrophages

**Figure 2:**
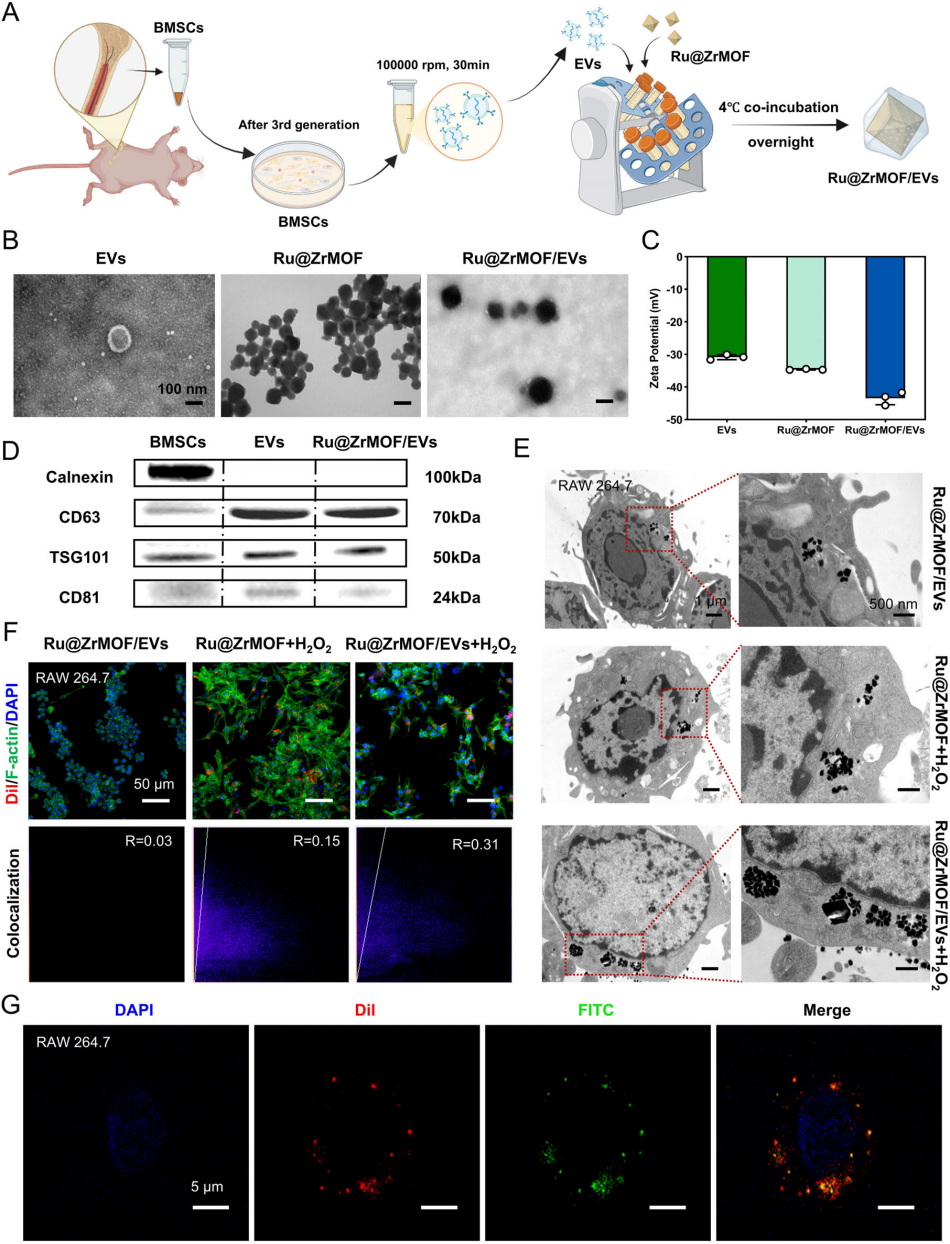
Construction of Ru@ZrMOF/EVs and targeting capacity to activated macrophages. A) Schematic illustration of the construction of Ru@ZrMOF/EVs. B) TEM image of the isolated EVs, Ru@ZrMOF, and Ru@ZrMOF/EVs. C) The Zeta potential of EVs, Ru@ZrMOF, and Ru@ZrMOF/EVs, *n* = 3 per group (three independent experiments, results are presented as means ± SD). D) WB analysis showing the expression of calnexin, CD63, TSG101, and CD81 expressed by BMSCs EVs and Ru@ZrMOF/EVs. E) Cellular uptake behavior in TEM images of the Ru@ZrMOF and Ru@ZrMOF/EVs in different treatment (no treatment or H_2_O_2_‐activated) of RAW264.7 cells. F) Cellular uptake behavior of different groups in CLSM images and fluorescence colocalization analysis (Green: F‐actin, Red: Dil, Blue: DAPI). G) CLSM images of Ru@ZrMOF/EVs. (Green: FITC labeled Ru@ZrMOF, Red: Dil labeled EVs).

Building upon the confirmed catalase‐mimetic activity of Ru@ZrMOF biocatalysts described in preceding sections, we developed an engineered EVs hybrid system through strategic biomembrane functionalization. **Figure** **2**A schematically illustrates the fabrication process of Ru@ZrMOF/EVs. Bone marrow‐derived mesenchymal stem cells (BMSCs) were prioritized as EV donors based on their documented therapeutic advantages, including low immunogenic potential and multipotent differentiation capacity.^[^ ^41^ ^]^ Following standardized protocols, primary murine BMSCs were expanded in culture and subsequently induced toward trilineage differentiation (osteogenic, chondrogenic, and adipogenic pathways) to confirm stem cell functionality. EV isolation was performed through differential ultracentrifugation with purity verification. TEM analysis confirmed successful EV coating on the Ru@ZrMOF surface (Figure 2B), while NTA revealed a median diameter of EV and Ru@ZrMOF/EVs are 99.5 and 173.1 nm, respectively (Figures S4 and S5, Supporting Information), and zeta‐potentials of EVs, Ru@ZrMOF and Ru@ZrMOF/EVs were −30.83 ± 0.79, −34.63 ± 0.16, and −43.45 ± 2.01 mV, respectively (Figure 2C). The more negative zeta‐potential of Ru@ZrMOF/EVs compared to the individual components further supported the successful EV coating. Notably, the membrane fusion process between negatively charged nanoparticles and EVs was achieved via optimized co‐incubation parameters.^[^ ^42^, ^43^ ^]^ The current understanding is that mechanisms regulating EV cellular uptake involve protein interactions that enable endocytosis.^[^ ^44^ ^]^ Specifically, EV membrane proteins (e.g., tetraspanins CD81/CD63) can engage with cellular receptors through ligand‐receptor interactions, facilitating cellular uptake. These characteristic tetraspanins not only serve as EV biomarkers but also mediate biointerface interactions critical for nanoparticle delivery.^[^ ^45^ ^]^ To validate system integrity, Western Blot (WB) analysis confirmed the presence of EV‐specific markers (TSG‐101, CD63, and CD81) and the endoplasmic reticulum protein calnexin (used as a negative control for EVs) (Figures 2D; S6, Supporting Information). The WB bands showed that BMSCs expressed calnexin, while both EVs and Ru@ZrMOF/EVs expressed TSG‐101, CD63, and CD81 and were negative for calnexin. This demonstrates the successful isolation of EVs and coating of Ru@ZrMOF.

We then further tested the macrophage uptake of the Ru@ZrMOF/EVs, especially in H_2_O_2_‐activated macrophages. The concentration of 100 µm H_2_O_2_ in vitro was chosen according to the estimated physiological concentration during the inflammatory reaction. The TEM images show that in activated macrophages, the intracellular uptake of Ru@ZrMOF/EVs was higher than that of Ru@ZrMOF, and the activated macrophages exhibited significantly greater uptake of Ru@ZrMOF/EVs than the macrophages untreated with H_2_O_2_ (Figure 2E). The confocal laser scanning microscopy (CLSM) images were also used to verify the macrophage uptake, the Dil‐labeled Ru@ZrMOF/EVs are shown in red fluorescence, and the fluorescence colocalization of red and green fluorescence demonstrated the degree of overlap between nano‐hybrids and cells. The Ru@ZrMOF/EVs + H_2_O_2_ group has the most overlapping red and green fluorescence and the highest Pearson's R value was confirmed by fluorescence colocalization analysis by Image J (Figures 2F; S7, Supporting Information). These results indicated that Ru@ZrMOF/EVs resulted in enhanced recruitment to highly activated macrophages. To further verify the successful construction and targeting capacity of Ru@ZrMOF/EVs, we labeled the Ru@ZrMOF with fluorescein isothiocyanate (FITC) and EVs with Dil. The CLSM result showed that Ru@ZrMOF was successfully coated by EVs and was endocytosed by murine mononuclear macrophage cells (RAW 264.7) (Figure 2G). The results demonstrate the successful construction of Ru@ZrMOF/EVs, with the EVs maintaining membrane integrity and not interfering with the cargo. This may enhance the therapeutic potential of EVs.

### 2.3 Ru@ZrMOF/EVs Cell Biocompatibility and ROS‐Scavenging Capacity

**Figure 3:**
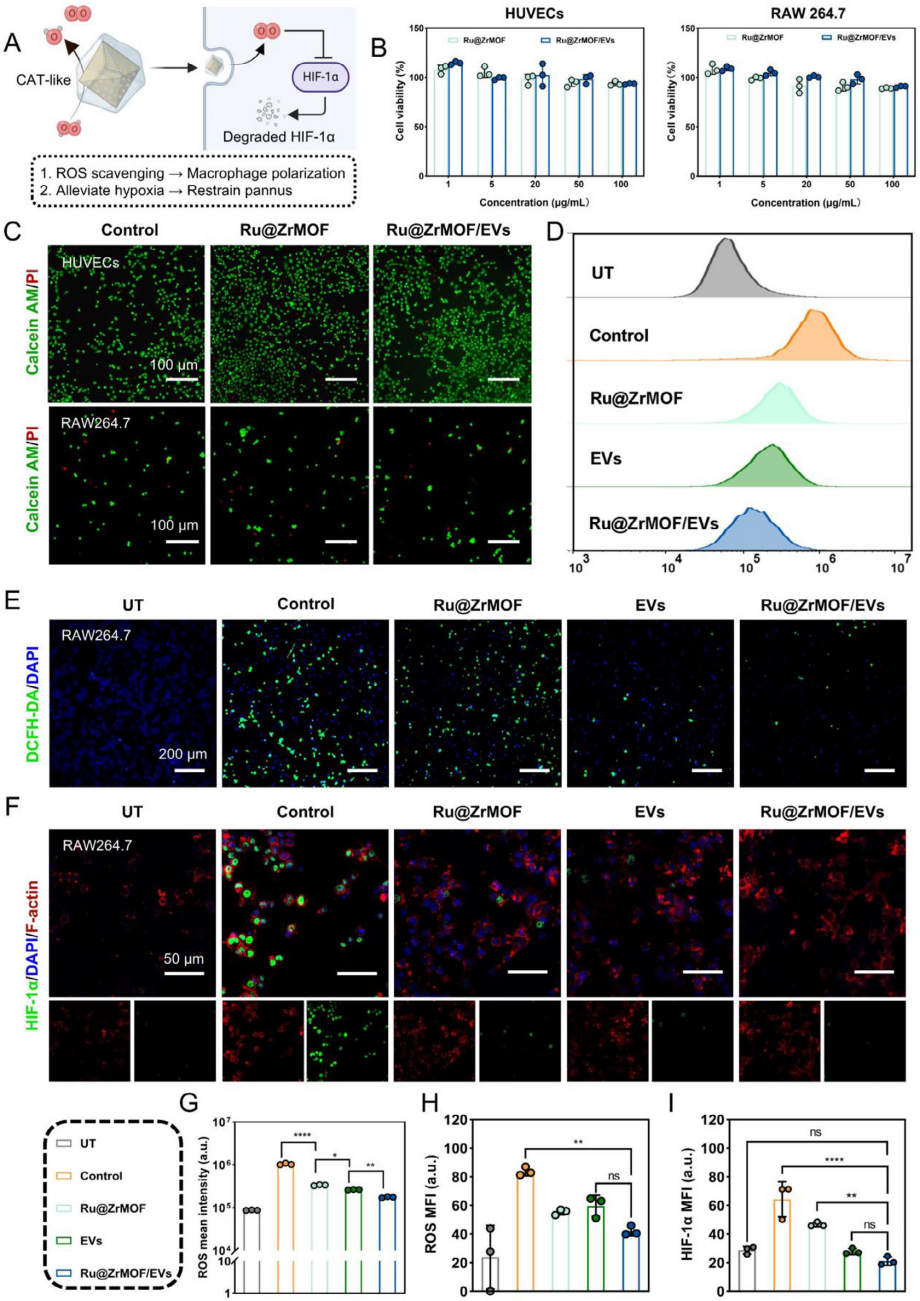
Ru@ZrMOF/EVs cell biocompatibility and ROS‐scavenging capacity. A) Schematic illustration of the mechanism of Ru@ZrMOF/EVs in cells. B) Viability of HUVECs and RAW 264.7 after incubation with Ru@ZrMOF/EVs at different concentrations. C) Representative images of Calcein‐AM/PI dual‐fluorescence stained HUVECs and RAW 264.7 at different groups, (green: live, red: dead). D) The ROS scavenging inside RAW 264.7 by flow cytometry and E) fluorescence images, and G) quantification of ROS mean intensity. H) MFI of DCFH‐DA staining. F) HIF‐1α in RAW264.7 (green: F‐actin, red: Dil, blue: DAPI), and analysis MFI in (I). In the above experiments, *n* = 3 per group (three independent experiments). Results are presented as means ± SD, *p < 0.05, **p < 0.01, ***p < 0.001, ****p < 0.0001, ns represents no significant difference; statistical significance was calculated using one‐way ANOVA followed by Tukey's post‐hoc test for multiple comparisons, all tests were two‐sided.

Having established that Ru@ZrMOF/EVs functions as an effective CAT‐like material with potent oxygen production and ROS scavenging abilities, we further took a comprehensive assessment of its capacity in cellular. **Figure** **3**A illustrates the mechanism of ROS‐scavenging and hypoxia alleviation of Ru@ZrMOF/EVs. Initially, we evaluated the cellular biocompatibility on human venous epithelial vascular cells (HUVECs) and RAW264.7., which were treated with varying concentrations of Ru@ZrMOF and Ru@ZrMOF/EVs. The results indicate high cell viability above 80%, suggesting that the Ru@ZrMOF/EVs have low cytotoxicity even at high concentrations (100 µg mL^−1^) (Figure 3B). To determine subsequent experimental concentrations, fluorescence microscopy images showing the effect of Ru@ZrMOF and Ru@ZrMOF/EVs on cell viability in HUVECs and RAW264.7 cells, the green fluorescence (Calcein AM) indicates live cells, while the red fluorescence (PI) marks dead cells. The result indicates that under concentrations of 20 µg mL^−1^, Ru@ZrMOF and Ru@ZrMOF/EVs have no significant difference in on cell viability (Figures 3C; S8–S10, Supporting Information). Besides, the HUVECs and RAW 264.7 Calcein AM fluorescence test by flow cytometry shows Ru@ZrMOF and Ru@ZrMOF/EVs exhibited no obvious cytotoxicity (Figure S11, Supporting Information). The hemolytic ratio is lower than 5% even at 1000 µg mL^−1^ of Ru@ZrMOF and Ru@ZrMOF/EVs (Figure S12, Supporting Information), thus indicating good blood cell compatibility.

Macrophage polarization to an active type, which is a well‐accepted pro‐inflammatory in vitro model, can be stimulated by H_2_O_2_ in a ROS‐dependent manner. In this study, we employed this model to evaluate the effect of Ru@ZrMOF/EVs on RAW264.7. 2,7‐dichlorofluorescein diacetate (DCFH‐DA) is applied as the fluorescence probe to explore intracellular ROS levels, as illustrated in Figure 3D,G, the Ru@ZrMOF/EVs group shows lower ROS intensity than other groups by flow cytometry. And the fluorescence microscopy assay gives similar results, the green fluorescence reflected the ROS level, was most obvious in the control group, which indicated that the H_2_O_2_ could induce the production of large amounts of ROS in RAW264.7 cells. In comparison with other groups, a great deal of ROS was scavenged in the Ru@ZrMOF/EVs group (Figure 3E,H). The H_2_O_2_ and hypoxic environment could induce RAW264.7 cells to upregulate the expression of HIF‐1α, which could be effectively inhibited by the treatment (Figure 3F,I). Therefore, we confirmed that the Ru@ZrMOF/EVs could effectively scavenge ROS, ameliorate hypoxia in cells, and downregulate the expression of HIF‐1α, which is an important nuclear transcription regulation factor adapting hypoxia.

### 2.4 Anti‐Inflammatory Response by Ru@ZrMOF/EVs

**Figure 4:**
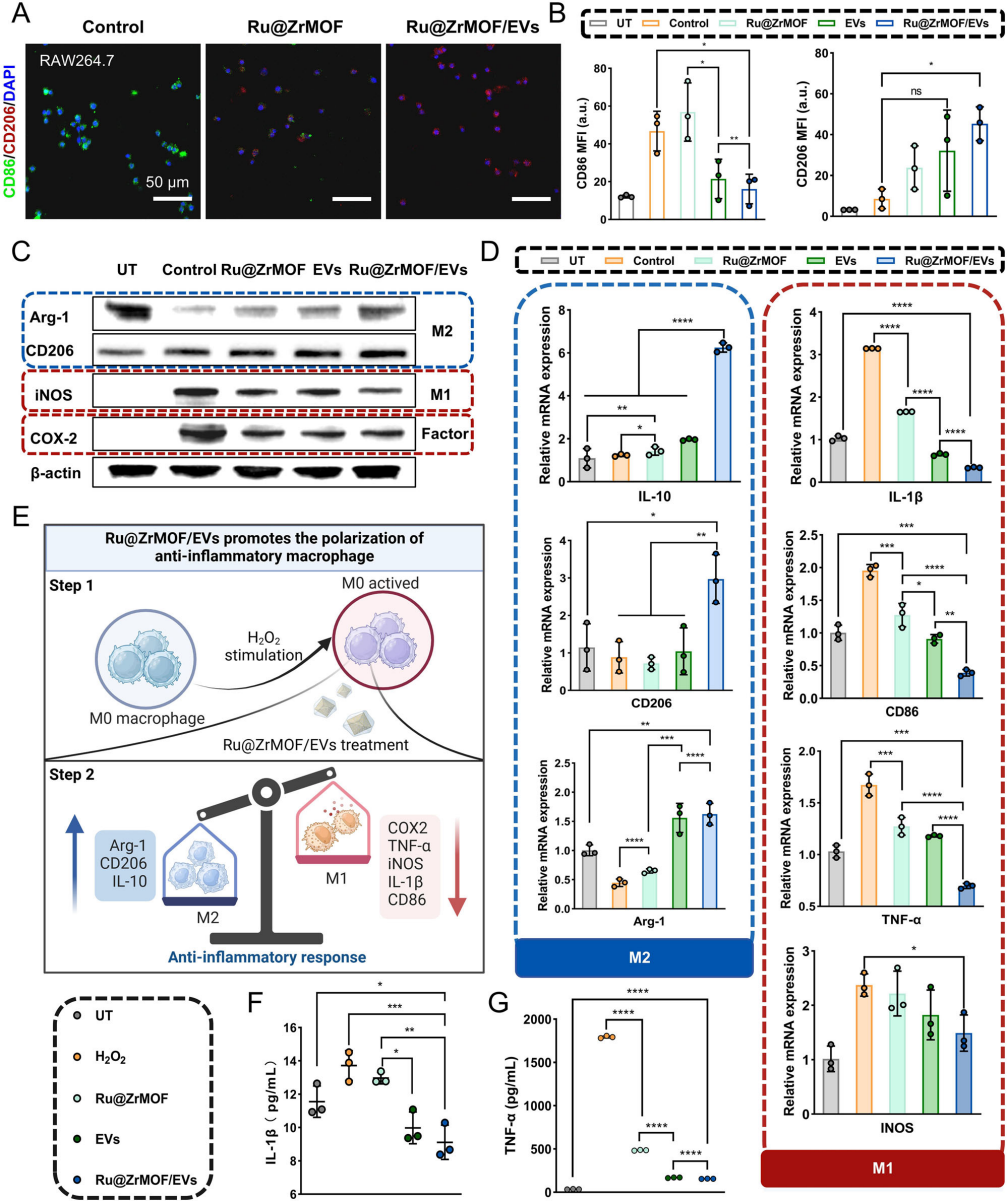
Anti‐inflammatory response by Ru@ZrMOF/EVs. A) Representative immunofluorescence images of CD86 (green) and CD206 (red) markers of RAW264.7 cells and B) semi‐quantitative analysis of the MFI. C) WB analysis of the M1 markers (iNOS), M2 markers (Arg‐1 and CD206) and the inflammatory factor markers (COX‐2). D) The IL‐10, CD206, Arg‐1, IL‐1β, CD86, TNF‐α, and iNOS gene expressions of RAW264.7 measured by RT‐qPCR. E) Schematic illustration of the mechanism of Ru@ZrMOF/EVs anti‐inflammatory response. F) IL‐1β and G) TNF‐α in cell supernatant measured by ELISA. In the above experiments, *n* = 3 per group (three independent experiments). Results are presented as means ± SD, *p < 0.05, **p < 0.01, ***p < 0.001, ****p < 0.0001, ns represents no significant difference; statistical significance was calculated using one‐way ANOVA followed by Tukey's post‐hoc test for multiple comparisons, all tests were two‐sided.

Immunofluorescence staining for CD86 (M1 marker) and CD206 (M2 marker) was used to examine macrophage polarization (**Figures** **4**A,B; S13, Supporting Information). After stimulation with H_2_O_2_, the fluorescence signal of CD86 was significantly enhanced, which could be effectively inhibited by Ru@ZrMOF, EVs, and Ru@ZrMOF/EVs groups, with the latter exhibiting the most predominant effect in M1 polarization suppression. Besides, Ru@ZrMOF/EVs not only demonstrated strong efficacy in inhibiting CD86 but also affected CD206 expression. Treatment with Ru@ZrMOF/EVs resulted in a significant increase in CD206, with Ru@ZrMOF/EVs showing superior enhancement compared to the control group. Therefore, these data suggest that Ru@ZrMOF/EVs are capable of modulating macrophage polarization. Specifically, WB bands reveal an increase in M2 markers (Arg‐1, CD206) and a decrease in M1 markers (iNOS), while also demonstrating a reduction in the inflammatory factor COX‐2 (Figures 4C; S14, Supporting Information). These findings further confirm the anti‐inflammatory effects of the Ru@ZrMOF/EVs. Real‐time quantitative PCR (RT‐qPCR) analysis was conducted to assess the impact of Ru@ZrMOF/EVs on the expression levels of inflammatory cytokines. As depicted in Figure 4D, Ru@ZrMOF/EVs treatment notably suppressed the mRNA expression of pro‐inflammatory cytokines such as IL‐1β, CD86, TNF‐α, and iNOS. Concurrently, the expression of anti‐inflammatory M2 markers, including IL‐10, CD206, and Arg‐1, was significantly upregulated by Ru@ZrMOF/EVs. Furthermore, an enzyme‐linked immunosorbent assay (ELISA) was utilized to evaluate the influence of Ru@ZrMOF/EVs on pro‐inflammatory cytokine expression. Results indicated that Ru@ZrMOF/EVs treatment was particularly effective in reducing the levels of IL‐1β and TNF‐α (Figure 4F,G). Taken together, our results demonstrated that Ru@ZrMOF/EVs could inhibit M1 polarization and promote M2 polarization, while also downregulating the expression of pro‐inflammatory cytokines (Figure 4E). Our findings indicate that Ru@ZrMOF/EVs have a stronger therapeutic impact suppressing inflammatory expression and promoting M2 polarization compared to bare EVs and Ru@ZrMOF. This highlights that Ru@ZrMOF/EVs provide a more favorable immune environment for inflammatory decrease and protection of cartilage.

In view of macrophage phenotypic transformation and cytokine level alterations, further exploration of the regulatory mechanisms of inflammatory‐related signaling cascades such as NF‐κB, MAPK, or JAK‐STAT can be conducted, including events like NF‐κB p65 nuclear translocation and IκBα/p65 phosphorylation, MAPK activation kinetics and downstream transcription factor activation, as well as JAK2/STAT1/3 phosphorylation, to clarify the specific signaling pathways through which they act in the treatment of RA.^[^ ^46^ ^]^

### 2.5 In vivo Ru@ZrMOF/EVs Targeting and Antiarthritis Capacity

**Figure 5:**
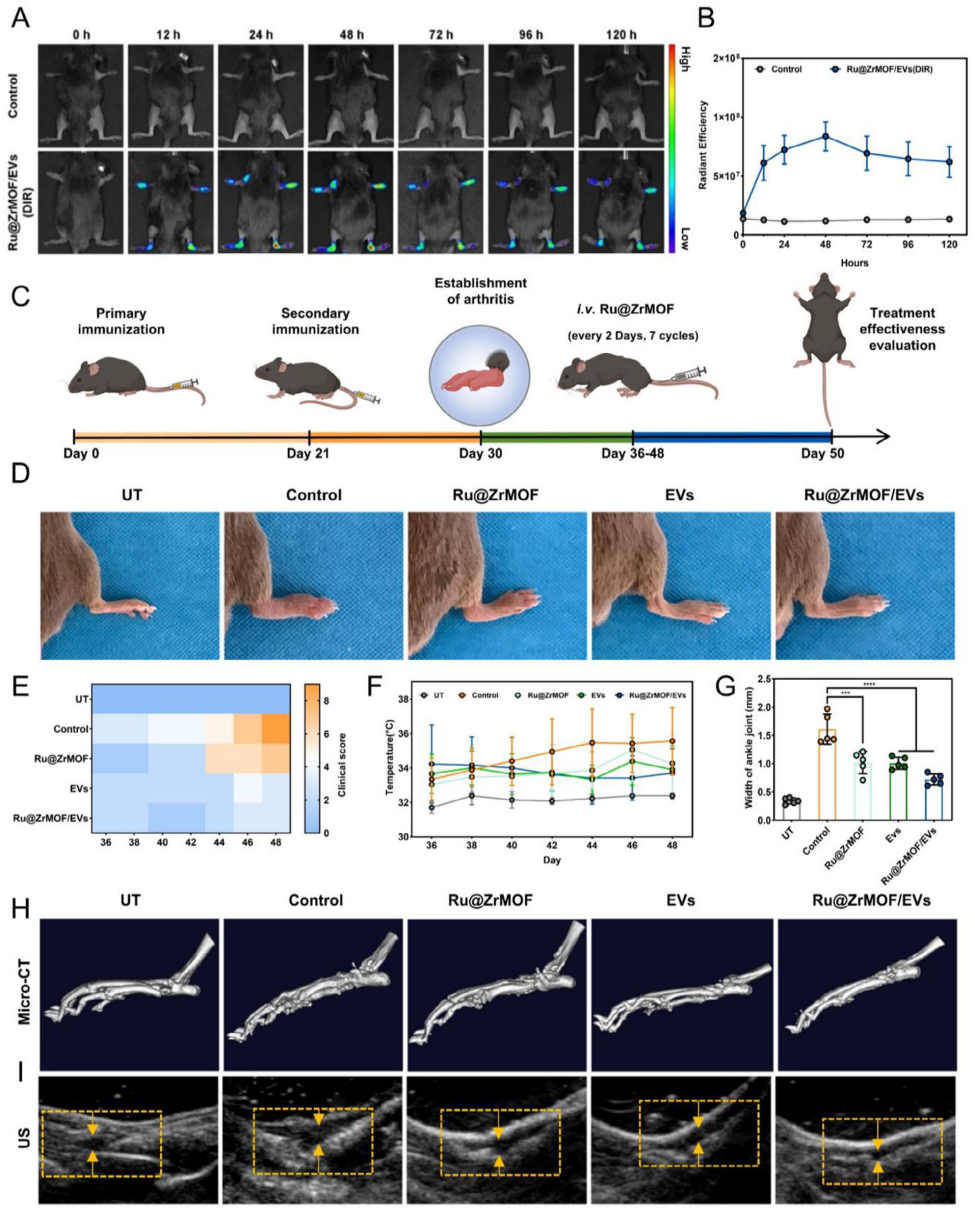
In vivo Ru@ZrMOF/EVs targeting and antiarthritis capacity. A) In vivo fluorescence images after intravenous injection of Ru@ZrMOF/EVs. B) Time‐dependent radiant efficiency curves for Ru@ZrMOF/EVs in the joint region for DIR. C) Treatment regimen in collagen‐induced arthritis mouse model (CIA). D) Representative photographs of the hind paws on day 50 after treatment in different groups. E) Hot map on visual arthritis score changes of CIA mice. F) Curves of hindlimb temperature variation in each group during the treatment. G) Analyze the width of ankle joint of (I). H) Micro‐CT and (I). High‐frequency US images of ankles in CIA mice after the last treatment. In (E–G), *n* = 5 per group (five independent experiments). Results are presented as means ± SD, *p < 0.05, **p < 0.01, ***p < 0.001, ****p < 0.0001; statistical significance was calculated using one‐way ANOVA followed by Tukey's post‐hoc test for multiple comparisons, all tests were two‐sided.

We used in vivo fluorescence images to observe the accumulation of Ru@ZrMOF/EVs in arthritic regions. As shown in **Figure** **5**A,B, fluorescence intensity in the joint area increases over time, peaking at 48 h post‐injection. The Ru@ZrMOF/EVs group exhibited significantly higher fluorescence intensity compared to the control group, which lacked fluorescence. After 120 h, we immediately executed the mice and took out the heart, liver, spleen, lungs, kidneys and limbs for *ex vivo* fluorescence imaging, and revealed the lung, liver and spleen as the primary site of Ru@ZrMOF/EVs accumulation, and the fluorescence intensity of the limbs of the Ru@ZrMOF/EVs group was significantly higher than that of the control group (Figure S15, Supporting Information). Meanwhile, we performed TEM observation of liver and ankle joints after 120 h of injection of Ru@ZrMOF group and Ru@ZrMOF/EVs group, and Figure S16 (Supporting Information) demonstrates more nanoparticle accumulation in both the liver and ankle joints of the Ru@ZrMOF/EV group compared to the Ru@ZrMOF group. The above experiments confirmed that Ru@ZrMOF/EVs modification 1) enhances targeted accumulation in the joint region; 2) aggregation peaked at 48 h after tail vein injection, suggesting a treatment interval of two days; 3) treatment sustained effects for up to 120 h. Figure 5C illustrates the process of establishing arthritis and the therapeutic regimen. Ankle joint redness and swelling objectively indicate the severity of collagen‐induced arthritis model (CIA) in mice after different groups of treatments. As exhibited in the photographs of Figure 5D, at the end of the treatment, the hind paws of the mice in Ru@ZrMOF/EVs group were significantly decongested compared with control group. The arthritis scores (Figure 5E) and paw thickness (Figure S17, Supporting Information) measured during the treatment verified that the effectiveness of Ru@ZrMOF/EVs group treatment was the most obvious. Joint inflammation in mice can be observed through the local temperature of the joints. The control group showed an increasing trend in the temperature of the hind limbs during the treatment process, whereas the temperature of the joints of the hind limbs of the Ru@ZrMOF/EVs group showed a decreasing trend, which was basically the same as that of the normal body surface temperature at the end of the treatment cycle on day 46 (Figure S18, Supporting Information; Figure 5F). Therefore, in the general observation, we found that the Ru@ZrMOF/EVs group was effective in reducing the symptoms of redness, swelling, and fever in the joints of mice. Micro‐computed tomography (Micro‐CT) images revealed differences in bone architecture among groups. In the control group, serious bone erosion or osteophytes were observed on the surface of bone cortexes. In comparison, the Ru@ZrMOF/EVs group appeared to exhibit a more intact bone surface, suggesting a potential protective effect against structural damage (Figure 5H). However, it is important to note that the micro‐CT analysis in this study was qualitative; future studies with calibrated scans are warranted to confirm these observations using quantitative morphometric parameters such as BV/TV or BMD. Furthermore, to evaluate the formation of inflammatory pannus—a hallmark pathological tissue characterized by synovial hyperplasia and neovascularization that drives joint destruction in RA—we performed high‐frequency ultrasonography to measure synovial thickness. The Ru@ZrMOF/EVs group demonstrated the thinnest synovium, which was significantly reduced compared to the control group, indicating a potent inhibition of pannus formation and anti‐inflammatory efficacy (Figure 5G,I).

Throughout the therapeutic regimen, all experimental subjects exhibited stable physiological parameters, with body mass variations remaining within ±5% of baseline values (Figure S19, Supporting Information). Serum biochemical analysis confirmed the absence of hepatorenal toxicity, as evidenced by comparable alanine aminotransferase (ALT), aspartate aminotransferase (AST), blood urea nitrogen (BUN), and creatinine (CREA) levels across treatment groups (Figure S20, Supporting Information). Subsequent histopathological evaluation via hematoxylin and eosin (H&E) staining demonstrated preserved tissue architecture in vital organs (cardiovascular, hepatic, splenic, pulmonary, and renal systems), with no observable necrosis, inflammatory infiltration, or structural abnormalities (Figure S21, Supporting Information). It's worth noting that the spleen H&E revealed a notable increase in megakaryocytes in the Ru@ZrMOF/EVs group compared to the UT group (Figure S21, Supporting Information). We interpret this as a positive systemic response rather than an adverse effect. Given that persistent inflammation in RA suppresses bone marrow hematopoiesis,^[^ ^47^ ^]^ the observed extramedullary megakaryopoiesis in the spleen likely signifies a compensatory recovery of hematopoietic function, highlighting the spleen's capacity, as a major extramedullary organ, to support platelet production in response to hematopoietic demand,^[^ ^48^ ^]^ following the successful alleviation of systemic inflammation by Ru@ZrMOF/EVs. This rebalancing of the hematopoietic‐immune axis, facilitated by the anti‐inflammatory and immunomodulatory functions of megakaryocytes (e.g., through IL‐10 secretion),^[^ ^49^ ^]^ may further contribute to the long‐term improvement of RA pathology.″

The comprehensive biosafety profile substantiates Ru@ZrMOF/EVs as a biocompatible nanotherapeutic platform with favorable systemic tolerance for rheumatoid arthritis intervention.

### 2.6 Evaluation of the Ru@ZrMOF/EVs Effectiveness of Joint Treatment In Vivo

**Figure 6:**
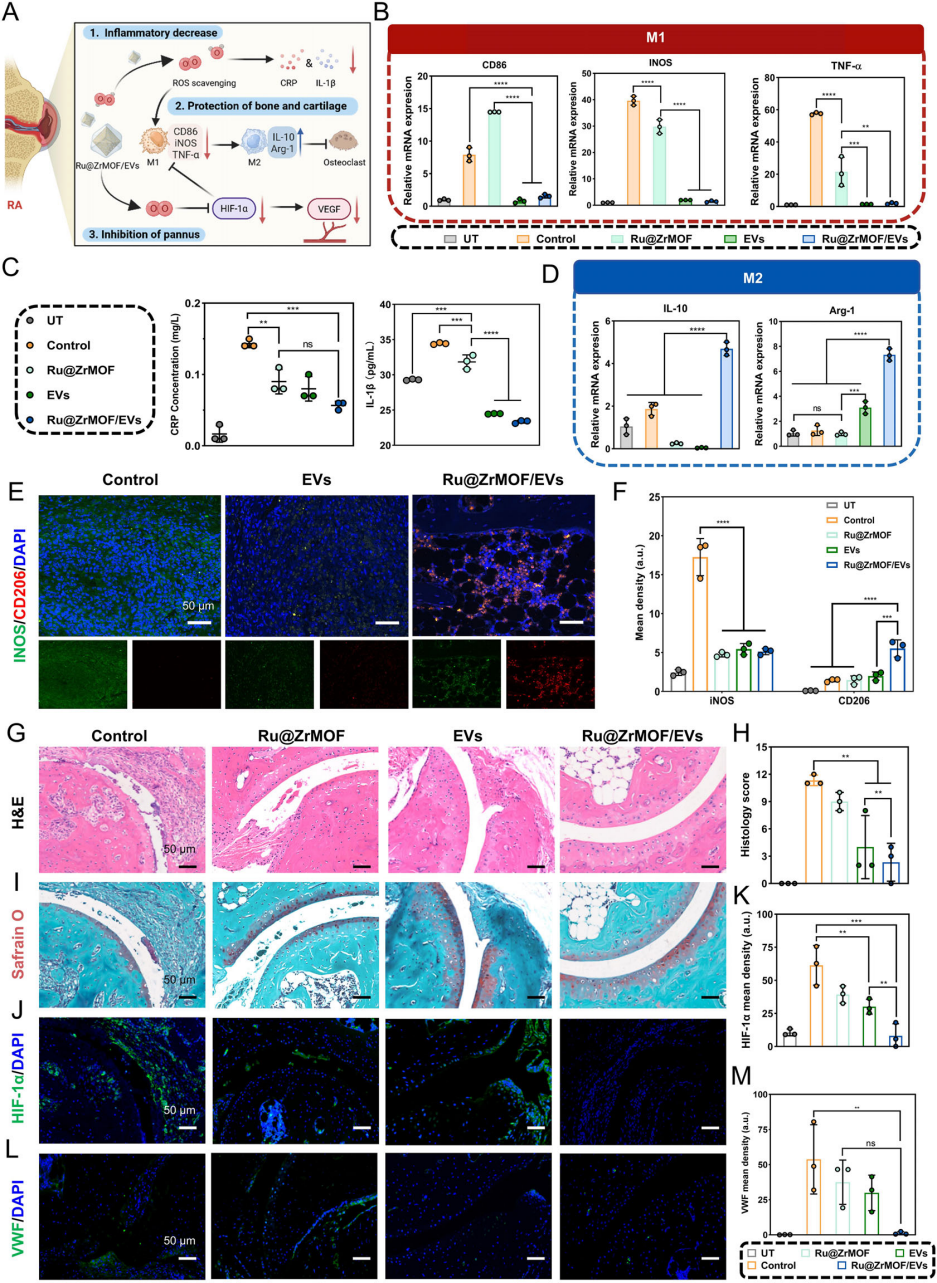
Evaluation of the Ru@ZrMOF/EVs effectiveness of joint treatment in vivo. A) Mechanism illustration of the Ru@ZrMOF/EVs treatment RA in vivo. B) Ankle joint tissue qRT‐PCR analysis of M1 markers of CD86, iNOS, TNF‐α, and D) M2 markers of IL‐10, Arg‐1. C) Detection of serum inflammatory indicators of CRP and IL‐1β concentrations. E) Images of iNOS (green) and CD206 (red) markers of RAW264.7 cells in the joint and F) semiquantitative analyzed of mean fluorescence intensity. G) Representative H&E‐stained images of ankle joint from all experimental groups. H) Histopathological scores of H&E. I) Representative Safranin O‐fast green staining images of ankle joint from all experimental groups. J) Representative immunofluorescent images of HIF‐1𝛼 and K) corresponding fluorescence mean density analysis. L) Representative immunofluorescent images of VWF and M) corresponding fluorescence mean density analysis. All scale bar = 50 *µm*. In (B–D,F,H,K,M) *n* = 3 per group (three independent experiments). Results are presented as means ± SD, *p < 0.05, **p < 0.01, ***p < 0.001, ****p < 0.0001, ns represents no significant difference; statistical significance was calculated using one‐way ANOVA followed by Tukey's post‐hoc test for multiple comparisons, all tests were two‐sided.

**Figure** **6**A shows the mechanism of Ru@ZrMOF/EVs treatment RA. The paw joint homogenates of M1 markers (CD86, iNOS, and TNF‐α) and M2 markers (IL‐10 and Arg‐1) were analyzed after sacrifice by PCR (Figure 6B,D). The results showed that M1 markers (CD86, iNOS, and TNF‐α) in the joint tissue of Ru@ZrMOF/EVs group were significantly lower than those in control group, while M2 markers (IL‐10 and Arg‐1) are higher. Serum concentrations of inflammatory cytokines CRP and IL‐1β decreased (Figure 6C). The phenotypic transition of macrophage markers in the synovial tissue of CIA mice was also observed and analyzed by immunofluorescence (Figures 6E,F; S22, Supporting Information) Therefore, we can conclude that the Ru@ZrMOF/EVs treatment effectively promoted the M1‐to‐M2 phenotypic transition in macrophages, and the reduction in CRP and IL‐1β levels not only suppressed the production of pro‐inflammatory cytokines but also facilitated macrophage phenotypic transition, which is crucial for tissue protection. Figure 6G–I and Figures S23 and S24 (Supporting Information) shows that the Ru@ZrMOF/EVs group exhibited the lowest symptoms and histopathological scores. In contrast, the control group had significant inflammatory cell infiltration, synovial hyperplasia, cartilage damage, and bone destruction, leading to higher scores. HIF‐1α accumulates in anoxic environments and is rapidly degraded in oxygen‐abundant environments. Meanwhile, some studies have indicated that CRP can upregulate the expression of HIF‐1α.^[^ ^46^, ^50^ ^]^ Thus, the CRP‐induced upregulation of HIF‐1α may promote hypoxic microenvironment‐driven inflammation and tissue destruction, thereby exacerbating the observed synovial hyperplasia and bone damage in the control group. Immunofluorescence staining revealed HIF‐1α expression in the joint tissues of mice (Figures 6J,K; S25, Supporting Information), compared with the control group, the fluorescence intensity decreased in all treatment groups, with the Ru@ZrMOF/EVs group showing the lowest fluorescence intensity. This suggests that the Ru@ZrMOF/EVs treatment effectively reduced the expression of HIF‐1α, indicating its potential role in modulating the hypoxic response and inflammation in the joint tissues. Pannus formation is one of the major pathological changes of RA, and the expression of von Willebrand factor (VWF) can be used to visualize and quantify vascular endothelium. The increased density of vWF‐positive structures in the control group (Figure 6L) is indicative of heightened vascularity, which is a hallmark of the angiogenic process within inflammatory pannus.^[^ ^51^, ^52^ ^]^ Additionally, the significant hypoxia in the arthritic area can stimulate the formation of local new blood vessels. Immunofluorescence staining was used to evaluate the expression of VWF and observe the angiogenesis of the joint capsule. The control group exhibited more new vessels, while the number of new vessels decreased in the other groups after treatment. (Figures 6L,M; S26, Supporting Information). These results indicated Ru@ZrMOF/EVs mitigating inflammation and hypoxia in joint tissues, further inhibiting new blood vessel formation, contributing to improved bone and cartilage protection.

Bare MOFs have limited biocompatibility and targeting, but encapsulating Ru@ZrMOF in EVs overcomes these: EVs improve biocompatibility and target the inflamed joint. Compared to EV therapy (low drug‐loading), MOF monotherapy (poor biodegradability), and traditional drug (systemic toxicity),^[^ ^53^ ^]^ Ru@ZrMOF/EVs accumulate more in inflamed joints and have lower toxicity. Though preclinical, the Ru@ZrMOF/EVs synergy offers a promising RA therapy paradigm balancing efficacy and safety.

## 3 Conclusion

In summary, we have successfully developed an EVs‐cloaked Ru@ZrMOF based on the MOF structure and its CAT‐mimicking biocatalytic properties. This innovative design integrates the high biocompatibility, targeting capabilities, and cartilage protection potential of EVs with the ROS‐scavenging and oxygen‐producing functions of Ru@ZrMOF, resulting in a potent therapeutic agent for RA. The treatment merits and therapeutic effects of Ru@ZrMOF/EVs have been systematically investigated, which can be included in the following stages: 1) targeting capacity and biocompatibility, which more uptake by activated macrophages and reduced cytotoxicity; 2) ROS scavenging capacity to alleviate joint inflammation by promoting the transformation of macrophages from the M1 to the M2 phenotype and reducing pro‐inflammatory factors, thereby mitigating bone erosion and promoting cartilage protection; 3) increased oxygen levels down‐regulate HIF‐1α, thereby alleviating RA progression by reducing cell oxygen deprivation and decreasing pannus formation, while HIF‐1α also functions to inhibit M1‐type macrophages. Therefore, we are convinced that this report on the nano‐biology hybrid Ru@ZrMOF/EVs for RA will promote the development of biocatalysts, presenting a novel possibility for the treatment of RA.

## 4 Experimental Section

### Preparation of Ru@ZrMOF and Extracellular Vesicles

First, synthesize UIO‐66‐NH_2_. 31.5 mg ZrCl_4_ and 24.5 mg H_2_BDC‐NH_2_ dissolved in 30 mL DMF (4 mL with HAC), sonication for 30 min and then heating at 120 (C for 3 h. After centrifuge (7000 rpm, 3 min), wash three times with DMF/ethanol (1/4 v/V) and dry. Then load Ru on UIO‐66‐NH_2_ (ZrMOF). Mass dispersion 1%Ru: 1 g UIO‐66‐NH_2_ and 10 mg RuCl_3_ dissolved in 100 mL of DI and stirred for 6 h. Add ascorbic acid 10 mg and stir for 6 h. After centrifuged, washed, and dried, the Ru@ZrMOF were collected for further assessments.

### Structural Characterization

Morphological features of ZrMOF and Ru@ZrMOF composites were analyzed by field‐emission scanning electron microscopy (FE‐SEM, Apreo S HiVac, Thermo Fisher Scientific) operating at 5 kV accelerating voltage. Crystalline phase identification was performed using X‐ray diffraction (XRD; Shimadzu XRD‐6100) with Cu Kα radiation (λ = 0.15406 nm, 40 kV/30 mA), employing a scanning rate of 5° min^−1^ across 5°–80° 2θ range. Surface chemical states were investigated through X‐ray photoelectron spectroscopy (XPS) on a K‐Alpha™+ system (Thermo Scientific) equipped with a hemispherical 180° dual‐focus analyzer and 128‐channel detector. Measurements utilized monochromatic Al Kα radiation (1486.6 eV) with 400 µm X‐ray spot size under ultrahigh vacuum (5 × 10^−^⁹ mbar). Specimen preparation involved compacting powdered samples onto conductive carbon tape mounted on standard SEM stubs, followed by 60‐s argon plasma cleaning to remove surface contaminants prior to analysis.

### Catalase‐Like H_2_O_2_ Catalytic Elimination Assay

A total of 10 mm of H_2_O_2_ and 50 µg mL^−1^ of biocatalysts (ZrMOF or Ru@ZrMOF) were mixed in PBS to 2 mL. Then, 50 µL of the solution was added to 100 µL of Ti(SO_4_)_2_ solution, including 319.2 mg of Ti(SO_4_)_2_ and 8.33 mL of H_2_SO_4_ in 51.33 mL of ultrapure water; the absorbance value was recorded every 5 min until 20 min. And after the reaction was completed, the absorbance of the solution at 405 nm was chosen to evaluate the remained H_2_O_2_ concentration. Additionally, Ru@ZrMOF with different concentrations (50, 100, 150, 200, and 250 µg mL^−1^) and 10 mm of H_2_O_2_ were mixed in PBS to show the correlation between concentration and H_2_O_2_ elimination.

### Catalase‐Like O_2_ Generation Assay

A total of 100 mm of H_2_O_2_ and 10 µg mL^−1^ of biocatalysts (ZrMOF or Ru@ZrMOF) were mixed in 20 mL of PBS, followed by measuring the O_2_ concentration using a dissolved oxygen meter (INESA, JPSJ‐605F) every 5 s until 250 s. Additionally, Ru@ZrMOF with different concentrations (10, 20, 30, 40, and 50 µg mL^−1^) was analyzed to show the correlation between concentration and O_2_ generation.

### Primary BMSCs Isolation and Characterization

Eight‐day‐old male neonatal Sprague–Dawley rats were chosen as the doners of primary BMSCs. After euthanasia, the femurs and tibias were separated from the surrounding tissue and washed by PBS with 2×penicillin‐streptomycin three times. Then, sterile scissors were used to remove the proximal and distal metaphysis and expose the bone cavity. A 1 mL syringe loaded with complete growth medium (α‐MEM with 10% FBS and 1× penicillin‐streptomycin) was used to rinse the intraosseous, and the fluids were collected by sterile tubes. After centrifugation at 300 g for 5 min, the BMSCs were resuspended by a complete growth medium and inoculated in culture flasks.

### BMSC‐Extracellular Vesicles (BMSC‐EVs) Isolation, Biocatalysts Construction, and Characterization

The BMSC‐EVs were isolated with ultracentrifuge methods. Briefly, the supernatant was collected following 300 g, 3 min, 4 (C, and another 2000 g, 20 min, 4 °C for separating the cells and debris. Then the supernatant was collected in Ultra‐Clear Centrifuge Tubes (344058, Beckman) following 16 500 rpm, 20 min, and 4 °C before transfer to new tube for 120 000 rpm, 120 min, and 4 °C using OPTIMA XPN‐100 Ultracentrifuge (Beckman). Finally, the BMSC‐EVs were resuspended by sterile PBS. The morphology of EVs was observed and recorded by TEM. The concentration and particle size distribution were measured by nanoparticle tracking analysis (NTA, ZETAVIEW). The Zeta potentials of EVs and EN were measured by NanoBrook Omni zeta potential analyzer.

For the construction of biocatalysts (Ru@ZrMOF‐cloaked with EVs, Ru@ZrMOF/EVs), 2 mg mL^−1^ Ru@ZrMOF was dissolved in 50 µL EVs cloaking solution with a concentration of 4.0–5.0 × 10^11^ EVs mL^−1^. Then the tubes were placed on the rotary mixer for overnight co‐incubation under 4 °C. Then, after centrifuging under 10 000 × g for 5 min, the Ru@ZrMOF/EVs were resuspended using sterile PBS. The morphology of the EVs and Ru@ZrMOF/EVs was visualized by TEM scanning. The expression of EVs‐specific markers (TSG‐101 and CD63) and negative markers (calnexin and CD81) were identified according to the standard Western Blot protocol.

The FITC‐ Ru@ZrMOF /Dil‐EVs particles were conjugated based on previous research. After centrifugation (5 min, 10 000 rpm) and washing with ethanol, the FITC‐ Ru@ZrMOF was collected. The Dil cell‐labeling solution (Invitrogen V22889) was used to label the BMSC‐EVs according to the protocol. After that, the FITC‐Ru@ZrMOF and Dil‐EVs were co‐incubated based on the abovementioned methods. The FITC‐EN/DiD‐EVs were then observed using confocal laser scanning microscopy (CLSM, N‐SIM S Nikon).

### Biocompatibility and Proliferation Assays

The human venous epithelial vascular cells (HUVECs) and RAW264.7 on the 96‐well plate at a density of 1 × 10^4^ per well. After 12 h, a gradient concentration of ZrMOF or Ru@ZrMOF was added to the corresponding group. After another 24 h, the cytotoxicity was evaluated by Cell Counting Kit‐8 (CCK‐8, HY‐K0301, MCE) according to the protocol.

### Calcein‐AM/PI Dual‐Fluorescence Staining

HUVECs or RAW264.7 were seeded on the 24‐well plate at a density of 5 × 10^4^ per well. And similar treatments mentioned above were applied. The Calcium/PI staining kit (C2015M, Beyotime) was used to demonstrate the live and dead cells that were observed and recorded by an inverted fluorescence microscope. The cell counts were calculated by ImageJ software.

### Detection of Intracellular ROS Scavenging

The cells were categorized into the following groups: 1) the untreated (UT) group (normal cells without any induction); 2) the control (Control) group (H_2_O_2_‐induced inflammatory model); 3) the Ru@ZrMOF group; 4) the EVs group; 5) the Ru@ZrMOF/EVs group (*n* = 3 per group). DCFH‐DA probe (D6883, Sigma, USA) was used to detect the intracellular ROS scavenging ability. RAW264.7 cells were seeded into 24‐well plates (each well containing 4 × 10^4^ cells) overnight. To establish an inflammatory model, cells were first stimulated with H_2_O_2_ (100 µm) for 4–6 h to induce oxidative stress and macrophage activation. Treatment with corresponding group wells and incubated for 24 h. Next, the cells were washed with PBS twice and added with DCFH‐DA solution (10 mm) prepared with serum‐free 1640. After washing with PBS, the ROS‐positive cells were observed and recorded under a fluorescence microscope, and the mean fluorescent intensity (MFI) was calculated using ImageJ software. The same method was used with flow cytometry fluorescent assay of the ROS.

### CD86 and CD206 Immunofluorescence Staining

The RAW264.7 cells were seeded onto 24‐well plates overnight at a density of 5 × 10^4^ per well. After the same treatments mentioned above, the cells were washed with sterile PBS twice and fixed with 4% paraformaldehyde. Then 0.5% Triton X‐100 was used to permeabilize the membrane for 10 min at room temperature. After PBS wash twice, 5% BSA was applied for blocking, and then the anti‐CD86 (1: 200, Proteintech) and anti‐CD206 (1: 200, Proteintech) antibodies were added for overnight incubation under 4 °C. After PBS washing, the goat anti‐mouse IgG (DyLight 594, Abbkine) and goat anti‐rabbit IgG (AF488, SAB) were added for 1 h incubation under 37 °C in the dark. Then, DAPI was added to label the nucleus for 5 min. After gentle PBS washing twice, the immunofluorescence staining was then observed and recorded under an inverted fluorescence microscope, and the mean fluorescence intensity was calculated by ImageJ software. And hypoxia inducible factor 1α in RAW264.7 cells after the different groups treatments and hypoxic environment induced was also detected by CLSM.

### Real‐Time Quantitative Polymerase Chain Reaction (RT‐qPCR)

After the corresponding interventions, the mRNA of the cells was extracted using Eastep™ Super Total RNA Extraction Kit (Promega, LS1040) according to the protocol. Then, the mRNA was reverse transcribed into cDNA using Hifair® III 1st Strand cDNA Synthesis SuperMix for qPCR (YEASEN, 11141ES10). Next, the Hieff UNICON® Universal Blue qPCR SYBR Green Master Mix was used for the RT‐qPCR detection. The RT‐qPCR process was run on QUANTSTUDIO3, Applied Biosystems.

### Establishment of Collagen‐Induced Arthritis Murine Model

All experimental procedures involving live animals were conducted in accordance with the institutional guidelines of Sichuan University and received ethical approval from the Animal Care Committee (Approval No. 20211354A). Seven‐to‐eight‐week‐old male DBA/1 mice were maintained under controlled environmental conditions (ambient temperature 22 ± 2 °C, relative humidity 50 ± 5%) with ad libitum access to standard rodent chow and reverse osmosis‐purified water, under a 12‐h photoperiod cycle. The CIA model was developed using the Chondrex protocol (Chondrex Inc., WA, USA) with modifications. The immunization protocol involved two‐stage administration: primary immunization with 200 µL bovine type II collagen solution (4 mg mL^−1^ in 0.1 M acetic acid) homogenized with an equal volume of complete Freund's adjuvant (CFA), followed by booster immunization 21 days later with antigen emulsified in incomplete Freund's adjuvant (IFA). Both emulsions were administered via intradermal injection at the caudal base using a 27‐gauge needle. Disease progression was assessed daily through clinical scoring based on established criteria: 1) Visible erythema and/or edema in at least two digital joints; 2) Impaired ambulation patterns; 3) Reduced food intake (>20% decrease from baseline). Successful model establishment required fulfillment of all three criteria with symptom persistence exceeding 72 h. Paw inflammation parameters were quantitatively monitored using digital caliper measurements of metatarsal joint diameter.

### In Vivo Arthritic Targeting Evaluation

To assess targeted biodistribution and retention kinetics, collagen‐induced arthritic mice were randomly allocated into two experimental cohorts: controls receiving equivalent PBS volumes and the Ru@ZrMOF/EVs treatment group. Following dual labeling with near‐infrared DiR and cyanine 5.5 (CY5.5), the nanoparticles were intravenously administered via the caudal vein. Serial fluorescence imaging was conducted at predetermined intervals (0, 12, 24, 46, 72, 96, and 120 h post‐injection) to monitor ankle joint signal accumulation. Quantitative analysis of time‐dependent fluorescence patterns was performed using Living Image® software (version 4.5.2, PerkinElmer), with region‐of‐interest (ROI) measurements focused on articular regions.

### In Vivo Treatment Regimen

Arthritic mice were divided into the UT (Untreated) group, control (CIA mice that received PBS injections), Ru@ZrMOF, EVs, and Ru@ZrMOF/EVs groups (*n* = 5). Furthermore, 100 µL of PBS, Ru@ZrMOF (2 mg mL^−1^), EVs (100 µL, the construction of EVs was based on 1 mL PBS per 50 µL EVs) and Ru@ZrMOF/EVs (100 µL, the construction of Ru@ZrMOF/EVs was based on 2 mg mL^−1^ Ru@ZrMOF per 50 µL EVs) were injected into the corresponding groups through the caudal veins every time. The treatment courses lasted seven cycles, every 2 days. Therapeutic efficacy was evaluated through joint score measurement, conventional evaluation of ankles, cytokine detection, and histological evaluation.

### Joint Score Measurement

Arthritis scores were established by the following criteria: 0 = no edema or arthritis, 1 = swelling in one type of joint, 2 = swelling in two types of joints, 3 = swelling in three types of joints, and 4 = swelling of the entire paw. The scores of all four limbs were added together to give a total score for each mouse, so the highest possible score was 16. The animals were scored every time before treatment and euthanasia.

### Imaging Assessment of Ankle Joint Pathology

Synovial membrane alterations were quantitatively analyzed using high‐resolution ultrasonography (7–15 MHz linear array transducer, Philips IU22 system, Netherlands), while osseous structural modifications were investigated through micro‐computed tomography (Quantum GX system, PerkinElmer, USA). Pre‐euthanasia ultrasound examinations were conducted in superficial tissue imaging mode with acoustic coupling gel ensuring optimal probe‐skin interface. Synovial membrane thickness measurements were systematically recorded at standardized anatomical positions. For 3D bone architecture evaluation, micro‐CT scanning was performed under standardized parameters: 80 kV tube voltage, 100 µA current, and 50 µm isotropic voxel resolution. Volume reconstruction and morphological analysis were executed using Analyze 12.0 software suite (PerkinElmer), generating comprehensive 3D renderings of trabecular bone microstructure. This dual‐modality approach enabled concurrent assessment of soft tissue inflammation and subchondral bone remodeling patterns.

### Histopathological Assessment of Murine Ankle Joints

Following dissection, mouse ankle specimens were fixed in 10% neutral buffered formalin and subsequently decalcified using 10% EDTA solution (pH 7.4). Paraffin‐embedded decalcified tissues were sectioned at 2.5 µm thickness for histological processing. Tissue sections underwent differential staining with hematoxylin‐eosin (H&E), Safranin O‐fast green, and immunohistochemical staining for HIF‐1α and von Willebrand factor (VWF), followed by systematic microscopic evaluation conducted under blinded conditions. Quantitative analysis of joint pathology was performed according to established scoring criteria based on H&E‐stained sections. Four distinct histopathological parameters were independently assessed using a 4‐tier grading system (0–3). Inflammatory infiltration: 0 – Absent; 1 – Focal leukocyte accumulation; 2 – Partial joint space occupation by inflammatory cells; 3 – Complete joint space filling. Synovial hyperplasia: 0 – Normal synovium; 1 – Mild synovial thickening (<3 cell layers); 2 – Moderate thickening (3–5 cell layers); 3 – Severe hyperplasia (>5 cell layers). Cartilage integrity: 0 – Intact surface; 1‐ Superficial fibrillation; 2 – Matrix loss extending to middle zone; 3 – Full‐thickness erosion. Bone remodeling: 0 – Normal architecture; 1 – Minor resorption pits; 2 – <30% trabecular loss; 3 – >30% structural damage. Cumulative pathological scores (maximum 12 points) were calculated through summation of individual parameter scores. All microscopic evaluations were performed using standardized brightfield/darkfield illumination with three independent verification.

### Statistical Analysis

Preprocessing procedures for data were performed prior to statistical analysis, including transformation of non‐normal data, normalization of variables to a common scale, and outlier detection using the interquartile range method. Experimental results were expressed as mean ± SD. The sample size (n) for each statistical analysis refers to the number of independent biological replicates, with specific values indicated in the figure legends (e.g., *n* = 3 represents three independent experiments). Statistical differences were assessed using one‐way analysis of variance (ANOVA) followed by Tukey's post‐hoc test for multiple comparisons, and two‐tailed Student's *t*‐test for pairwise comparisons. The significance level (alpha value) was set at 0.05. Prior to analysis, the assumptions for ANOVA (normality via Shapiro–Wilk test and homogeneity of variance via Levene's test) were verified, and data meeting these assumptions were included. Statistical significance was defined as *p < 0.05, **p < 0.01, ***p < 0.001, ****p < 0.0001, and ns represents no significant difference. All statistical analyses were performed using GraphPad Prism version 8.0 (GraphPad Software, Inc., CA, USA).

## Conflict of Interest

The authors declare no conflict of interest.

## Author Contributions

X.H.W., J.B.H., and S.W.Z. contributed equally to this work. X.H.W., J.B.H., S.W.Z., and F.X.D. performed the experiments and analyzed the results. S.W.Z., S.J.C., and F.X.D. assisted with the figure production and experimental design. X.H.W. and Y.J.T. wrote the manuscript. L.Q.Z., L.Q., and Y.J.T. designed the experiments, revised the manuscript, and supervised the whole project. All authors discussed the results and commented on the manuscript.

## Supporting information



Supporting Information

## Data Availability

The data that support the findings of this study are available in the supplementary material of this article.
