# Supplementary material for: Biocatalytic Nanoregulators Restore Joint Redox‐Immune Homeostasis in Rheumatoid Arthritis
Source: Adv Sci (Weinh). 2026 Jan 14;13(10):e02894. doi: 10.1002/advs.202502894 (PMC12915140; doi:10.1002/advs.202502894)
Supplement: Supplementary file 1 — Supporting Information [file ADVS-13-e02894-s001.docx]

Supplementary Information for

Biocatalytic Nanoregulators Restore Joint Redox-Immune Homeostasis in Rheumatoid Arthritis

*Xingheng Wang,^1,a^ Jianbo Huang,^1,a^ Shuwei Zhang,^1,a^ Sujiao Cao,^a^ Fangxue Du,^a^ Liqiang Zhou,*^,b^ Li Qiu,*^,a^ and* *Yuanjiao Tang *^,a^*

^a^ Department of Ultrasound, National Clinical Research Center for Geriatrics, Med-X Center for Materials, West China Hospital, Sichuan University, Chengdu, 610041, China

^b^ MOE Frontiers Science Center for Precision Oncology Faculty of Health Sciences University of Macau, Macau SAR 999078, China

^1^ These authors contributed equally to this work.

*** Corresponding authors: qiulihx@scu.edu.cn (L. Qiu); [liqiangzhou@um.edu.mo](mailto:liqiangzhou@um.edu.mo) (L.Q. Zhou); yuanjiaotang@foxmail.com (Y.J. Tang)

**Contents**

Supplymentary Experimental Section

Supplementary Figure 1 to 25 and Table 1

**Supplementary Experimental Section**

**Preparation of Ru@ZrMOF and extracellular vehicles.** First synthesize UIO-66-NH_2_. 31.5 mg ZrCl_4_ and 24.5 mg H_2_BDC-NH_2_ dissolved in 30 mL DMF (4 mL with HAC), sonication for 30 min and then heating at 120℃ for 3 h. After centrifuge (7000 rpm, 3 min), wash three times with DMF/ethanol (1/4 v/V) and dry. Then load Ru on UIO-66-NH_2_ (ZrMOF). Mass dispersion 1%Ru: 1g UIO-66-NH_2_ and 10 mg RuCl_3_ dissolved in 100 mL of DI and stirred for 6 h. Add ascorbic acid 10 mg and stir for 6 h. After centrifuged, washed and dried, the Ru@ZrMOF were collected for further assessments.

**Morphology characterization.** The morphologies of ZrMOF and Ru@ZrMOF were studied using scanning electron microscopy (SEM, Apreo S HiVoc, Thermo Fisher Scientific). The phase composition was identified by a Shimadzu XRD-6100 diffractometer using Cu Kα radiation (λ = 0.15406 nm). X-ray photoelectron spectroscopy (XPS) was performed to detect the valence states and electronic structures of ZrMOF and Ru@ZrMOF on K-Alpha™ + X-ray Photoelectron Spectrometer System (Thermo Scientific) with a Hemispheric 180° dual-focus analyzer and a 128-channel detector. X-ray monochromator is Micro focused Al-Kα radiation. For the measurement, the prepared powder samples were pressed and loaded on carbon taps, then pasted onto the sample holder for measurement. The data was collected with an X-ray spot size of 400 μm.

**Catalase-like H_2_O_2_ catalytic elimination assay.** A total of 10 mM of H_2_O_2_ and 50 μg/mL of biocatalysts (ZrMOF or Ru@ZrMOF) were mixed in PBS to 2 mL. Then, 50 μL of the solution was added to 100 μL of Ti(SO_4_)_2_ solution, including 319.2 mg of Ti(SO_4_)_2_ and 8.33 mL of H_2_SO_4_ in 51.33 mL of ultrapure water; the absorbance value was recorded every 5 min until 20 min. And after the reaction was completed, the absorbance of the solution at 405 nm was chosen to evaluate the remained H_2_O_2_ concentration. Additionally, Ru@ZrMOF with different concentrations (50, 100, 150, 200, and 250 μg/mL) and 10 mM of H_2_O_2_ were mixed in PBS to show the correlation between concentration and H_2_O_2_ elimination.

**Catalase-like O_2_ generation assay.** A total of 100 mM of H_2_O_2_ and 10 μg/mL of biocatalysts (ZrMOF or Ru@ZrMOF) were mixed in 20 mL of PBS, followed by measuring the O_2_ concentration using a dissolved oxygen meter (INESA, JPSJ-605F) every 5 s until 250 s. Additionally, Ru@ZrMOF with different concentrations (10, 20, 30, 40, and 50 μg/mL) was analyzed to show the correlation between concentration and O_2_ generation.

**Primary BMSCs isolation and characterization.** 8-day-old male neonatal Sprague-Dawley rats were chosen as the doners of primary BMSCs. After euthanasia, the femurs and tibias were separated from the surrounding tissue and washed by PBS with 2×penicillin-streptomycin three times. Then, sterile scissors were used to remove the proximal and distal metaphysis and expose the bone cavity. A 1 mL syringe loaded with complete growth medium (α-MEM with 10% FBS and 1× penicillin-streptomycin) was used to rinse the intraosseous, and the fluids were collected by sterile tubes. After centrifugation at 300 g for 5 minutes, the BMSCs were resuspended by a complete growth medium and inoculated in culture flasks.

**BMSC-extracellular vesicles (EVs) isolation, biocatalysts construction, and characterization**. The BMSC-EVs were isolated with ultracentrifuge methods. Briefly, the supernatant was collected following 300 g, 3 min, 4 ℃, and another 2000 g, 20 min, 4 ℃ for separating the cells and debris. Then the supernatant was collected in Ultra-Clear Centrifuge Tubes (344058, Beckman) following 16500 rpm, 20 min, and 4 ℃ before transfer to new tube for 120,000 rpm, 120 min, and 4 ℃ using OPTIMA XPN-100 Ultracentrifuge (Beckman). Finally, the BMSC-EVs were resuspended by sterile PBS. The morphology of EVs was observed and recorded by transmission electron microscopy (TEM). The concentration and particle size distribution were measured by nanoparticle tracking analysis (NTA, ZETAVIEW). The Zeta potentials of EVs and EN were measured by NanoBrook Omni zeta potential analyzer.

For the construction of biocatalysts (Ru@ZrMOF-cloaked with EVs, Ru@ZrMOF/EVs), 2 mg/mL Ru@ZrMOF was dissolved in 50 μL EVs cloaking solution with a concentration of 4.0-5.0 × 10^11^ EVs/mL. Then the tubes were placed on the rotary mixer for overnight co-incubation under 4 ℃. Then, after centrifuging under 10000×g for 5 min, the Ru@ZrMOF/EVs were resuspended using sterile PBS. The morphology of the EVs and Ru@ZrMOF/EVs was visualized by TEM scanning. The expression of EVs-specific markers (TSG-101 and CD63) and negative markers (calnexin and CD81) were identified according to the standard Western Blot protocol.

The FITC- Ru@ZrMOF /Dil-EVs particles were conjugated based on previous research.^[1]^ After centrifugation (5 min, 10,000 rpm) and washing with ethanol, the FITC- Ru@ZrMOF was collected. The Dil cell-labeling solution (Invitrogen V22889) was used to label the BMSC-EVs according to the protocol. After that, the FITC-Ru@ZrMOF and Dil-EVs were co-incubated based on the abovementioned methods. The FITC-EN/DiD-EVs were then observed using confocal laser scanning microscopy (CLSM, N-SIM S Nikon).

**Biocompatibility and proliferation assays.** The human venous epithelial vascular cells (HUVECs) and RAW264.7 on the 96-well plate at a density of 1×10^4^/well. After 12 hours, a gradient concentration of ZrMOF or Ru@ZrMOF was added to the corresponding group. After another 24 hours, the cytotoxicity was evaluated by Cell Counting Kit-8 (CCK-8, HY-K0301, MCE) according to the protocol.

**Calcein-AM/PI dual-fluorescence staining.** HUVECs or RAW264.7 were seeded on the 24-well plate at a density of 5×10^4^/well. And similar treatments mentioned above were applied. The Calcium/PI staining kit (C2015M, Beyotime) was used to demonstrate the live and dead cells that were observed and recorded by an inverted fluorescence microscope. The cell counts were calculated by ImageJ software.

**Detection of intracellular ROS scavenging.** The cells were categorized into the following groups: (1) the untreated (UT) group (normal cells without any induction); (2) the control (Control) group (H_2_O_2_-induced inflammatory model); (3) the Ru@ZrMOF group; (4) the EVs group; (5) the Ru@ZrMOF/EVs group (n = 3 per group). DCFH-DA probe (D6883, Sigma, USA) was used to detect the intracellular ROS scavenging ability. RAW264.7 cells were seeded into 24-well plates (each well containing 4 × 10^4^ cells) overnight. To establish an inflammatory model, cells were first stimulated with H_2_O_2_ (100 μM) for 4-6 hours to induce oxidative stress and macrophage activation. Treatment with corresponding group wells and incubated for 24 h. Next, the cells were washed with PBS twice and added with DCFH-DA solution (10 mM) prepared with serum-free 1640. After washing with PBS, the ROS-positive cells were observed and recorded under a fluorescence microscope, and the mean fluorescent intensity (MFI) was calculated using ImageJ software. The same method was used with flow cytometry fluorescent assay of the ROS.

**CD86 and CD206 immunofluorescence staining.** The RAW264.7 cells were seeded onto 24-well plates overnight at a density of 5×10^4^/well. After the same treatments mentioned above, the cells were washed with sterile PBS twice and fixed with 4% paraformaldehyde. Then 0.5% Triton X-100 was used to permeabilize the membrane for 10 min at room temperature. After PBS wash twice, 5% BSA was applied for blocking, and then the anti-CD86 (1: 200, Proteintech) and anti-CD206 (1 : 200, Proteintech) antibodies were added for overnight incubation under 4 ℃. After PBS washing, the goat anti-mouse IgG (DyLight 594, Abbkine) and goat anti-rabbit IgG (AF488, SAB) were added for 1 h incubation under 37 ℃ in the dark. Then, DAPI was added to label the nucleus for 5 min. After gentle PBS washing twice, the immunofluorescence staining was then observed and recorded under an inverted fluorescence microscope, and the mean fluorescence intensity was calculated by ImageJ software. And hypoxia inducible factor 1α in RAW264.7 cells after the different groups treatments and hypoxic environment induced was also detected by CLSM.

**Real-time quantitative Polymerase Chain Reaction (RT-qPCR).** After the corresponding interventions, the mRNA of the cells was extracted using Eastep™ Super Total RNA Extraction Kit (Promega, LS1040) according to the protocol. Then, the mRNA was reverse transcribed into cDNA using Hifair® Ⅲ 1st Strand cDNA Synthesis SuperMix for qPCR (YEASEN, 11141ES10). Next, the Hieff UNICON® Universal Blue qPCR SYBR Green Master Mix was used for the RT-qPCR detection. The RT-qPCR process was run on QUANTSTUDIO3, Applied Biosystems.

**Establishment of the** **collagen-induced arthritis (CIA) mouse model.** All experimental procedures involving live animals were conducted in accordance with the institutional guidelines of Sichuan University and received ethical approval from the Animal Care Committee (Approval No. 20211354A). Seven-to-eight-week-old male DBA/1 mice were maintained under controlled environmental conditions (ambient temperature 22 ± 2°C, relative humidity 50 ± 5%) with ad libitum access to standard rodent chow and reverse osmosis-purified water, under a 12-hour photoperiod cycle. The CIA model was developed using the Chondrex protocol (Chondrex Inc., WA, USA) with modifications. The immunization protocol involved two-stage administration: primary immunization with 200 µL bovine type II collagen solution (4 mg/mL in 0.1M acetic acid) homogenized with an equal volume of complete Freund's adjuvant (CFA), followed by booster immunization 21 days later with antigen emulsified in incomplete Freund's adjuvant (IFA). Both emulsions were administered via intradermal injection at the caudal base using a 27-gauge needle. Disease progression was assessed daily through clinical scoring based on established criteria: 1) Visible erythema and/or edema in at least two digital joints; 2) Impaired ambulation patterns; 3) Reduced food intake (>20% decrease from baseline). Successful model establishment required fulfillment of all three criteria with symptom persistence exceeding 72 hours. Paw inflammation parameters were quantitatively monitored using digital caliper measurements of metatarsal joint diameter.

**Arthritic targeting *in vivo*.** To observe the targeting effect and long-time retention, the arthritic mice were divided into control and Ru@ZrMOF/EVs groups. After staining with DiR and CY5.5, the nanoparticles were injected through the caudal veins. An equal volume of PBS was injected into the control group. The fluorescence intensities at the ankles were observed at 0, 12, 24, 46, 72, 96 and 120 h after the injection by *in vivo* fluorescence imaging. Afterward, the Living Image software was used to analyze the fluorescence intensities at different time points.

***In vivo* treatment regimen.** Arthritic mice were divided into the UT (Untreated) group, control (CIA mice that received PBS injections), Ru@ZrMOF, EVs, and Ru@ZrMOF/EVs groups (n = 5). Furthermore, 100 μL of PBS, Ru@ZrMOF (2 mg/mL), EVs (100 μL, the construction of EVs was based on 1 mL PBS per 50 μL EVs) and Ru@ZrMOF/EVs (100 μL, the construction of Ru@ZrMOF/EVs was based on 2 mg/mL Ru@ZrMOF per 50 μL EVs) were injected into the corresponding groups through the caudal veins every time. The treatment courses lasted 7cycles, every two days. Therapeutic efficacy was evaluated through joint score measurement, conventional evaluation of ankles, cytokine detection, and histological evaluation.

**Joint score measurement.** Arthritis scores were established by the following criteria: 0 = no edema or arthritis, 1 = swelling in one type of joint, 2 = swelling in two types of joints, 3 = swelling in three types of joints, and 4 = swelling of the entire paw. The scores of all four limbs were added together to give a total score for each mouse, so the highest possible score was 16. The animals were scored every time before treatment and euthanasia.

**Conventional imaging evaluation of ankles.** A high-frequency ultrasound was used to evaluate the synovitis, and the Micro-CT was used to evaluate the bony changes in the ankles. Ultrasound examinations were performed before the animals were sacrificed with an ultrasonic device (IU22, Philips, Amsterdam, Netherlands). The probe frequency was 7~15 MHz, and the superficial condition was selected. The probe surface was covered with an ultrasound gel to enable total contact with the skin. Additionally, the synovial thickness of the ankles was measured. As for the Micro-CT scanning, the Quantum GX Micro-CT Imaging System (PerkinElmer; MA, USA) was used, and the scanning parameters were voltage, 80 kV; current, 100 μA; pixel size, 50 μm. Analyze 12.0 software (PerkinElmer; MA, USA) was used to reconstruct 3D images, thereby visually representing the results.

**Histological evaluation of ankles.** The ankles of the mice were dissected and fixed in 10% formalin and decalcified in 10% neutral ethylene diamine tetraacetic acid solution. Decalcified tissue was embedded in paraffin. Moreover, the sections (2.5 mm) were stained with hematoxylin and eosin (H&E), Safranin O-fast green, HIF-1α and von Willebrand factor (VWF) and later subjected to light or dark microscopic examination in a blinded manner. Histopathological changes of the ankles were scored from 0 to 3 individually for inflammatory cell infiltration, pannus formation, synovial hyperplasia, articular cartilage damage, and bone destruction according to the previous study based on H&E staining. The grading scheme was based on the following scales: inflammatory scores (0 = no inflammatory cell infiltrate; 1 = a few inflammatory cell infiltrate; 2 = a part of the joint cavity filled with inflammatory cells; 3 = all of the joint cavity filled with inflammatory cells), synovitis scores (0 = healthy; 1 = mild thickening of the synovium; 2 = substantial thickening of the synovium; 3 = severe thickening of the synovium), cartilage damage scores (0 = normal; 1 = minor destruction of the cartilage surface; 2 = clear loss of cartilage; 3 = cartilage almost absent in the whole joint), and bone destruction scores (0 = normal; 1 = minor signs of destruction; 2 = up to 30 % destruction; 3 = more than 30 % destruction). A total score was obtained by adding those four scores, so the highest possible score was 12.

**Statistical Analysis.** Preprocessing procedures for data were performed prior to statistical analysis, including transformation of non-normal data, normalization of variables to a common scale, and outlier detection using the interquartile range method. Experimental results were expressed as mean ± SD. The sample size (n) for each statistical analysis refers to the number of independent biological replicates, with specific values indicated in the figure legends (e.g., n=3 represents three independent experiments). Statistical differences were assessed using one-way analysis of variance (ANOVA) followed by Tukey’s post-hoc test for multiple comparisons, and two-tailed Student’s t-test for pairwise comparisons. The significance level (alpha value) was set at 0.05. Prior to analysis, the assumptions for ANOVA (normality via Shapiro-Wilk test and homogeneity of variance via Levene’s test) were verified, and data meeting these assumptions were included. Statistical significance was defined as **p* < 0.05, ***p* < 0.01, ****p* < 0.001, *****p* < 0.0001, and ns represents no significant difference. All statistical analyses were performed using GraphPad Prism version 8.0 (GraphPad Software, Inc., CA, USA).

**
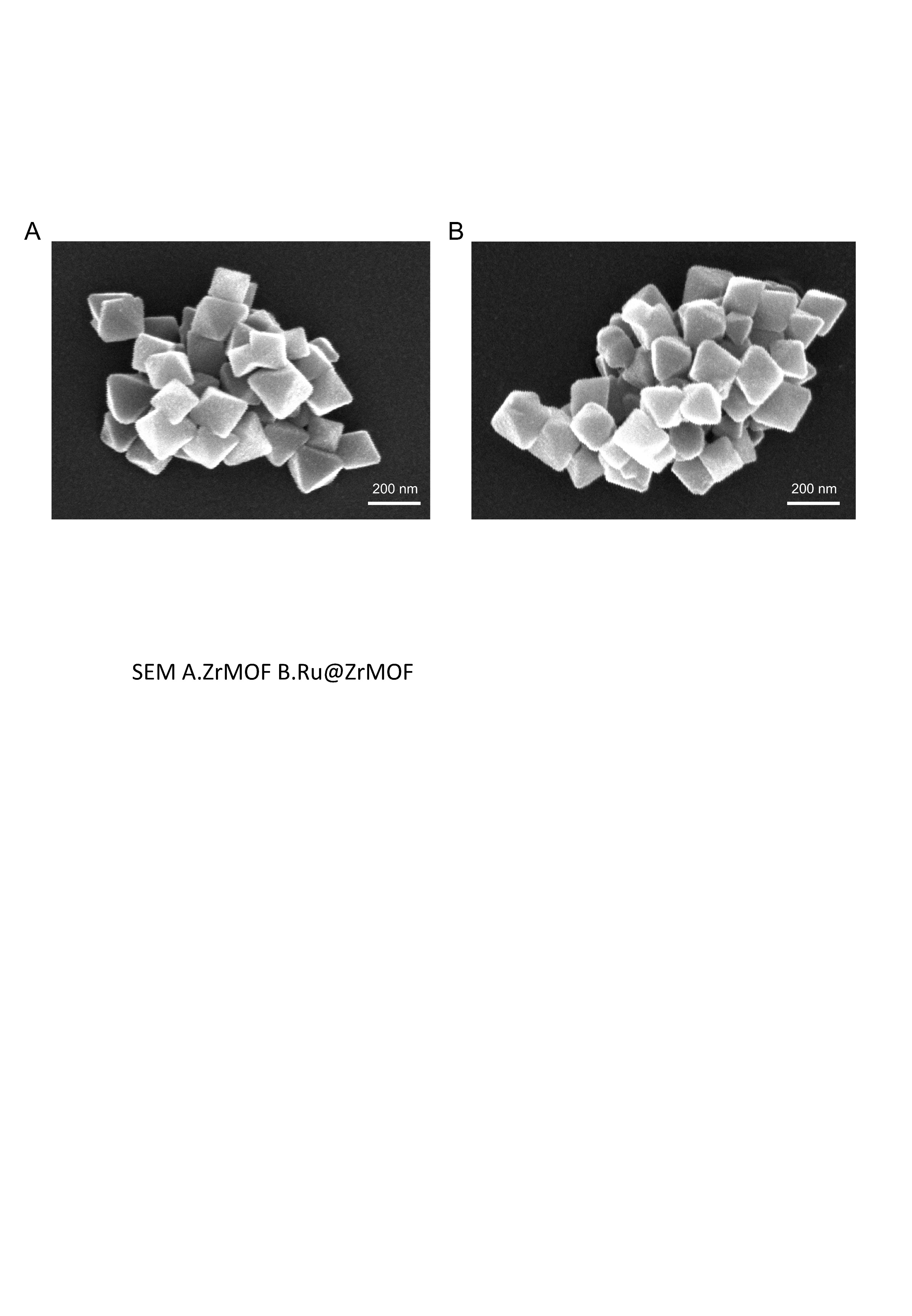
**

**Figure S1.** SEM of A) ZrMOF, B) Ru@ZrMOF.

**
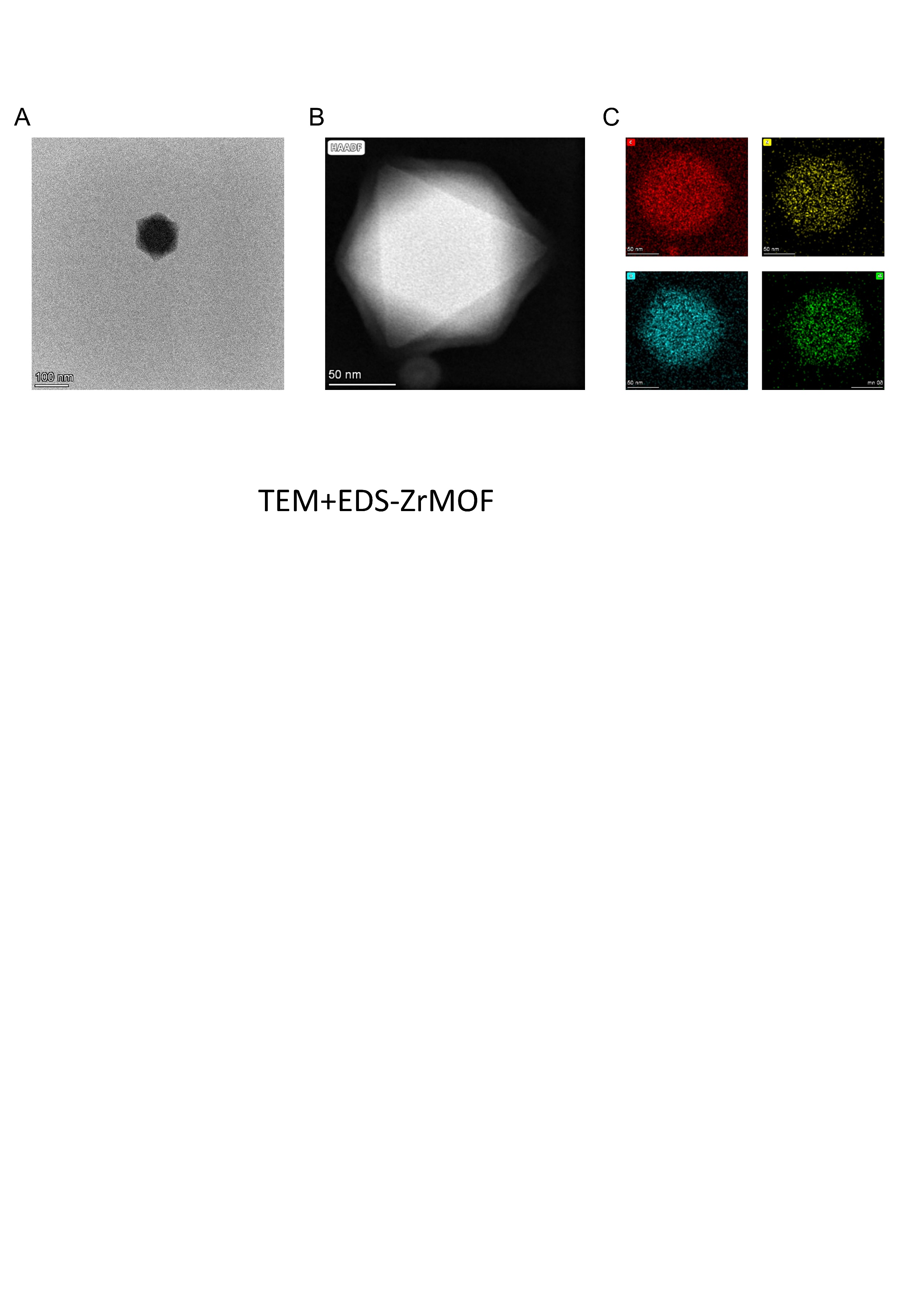
**

**Figure S2.** A) TEM, B) high angle annulardark field-scanning transmission electron microscopy (HAADF-TEM), C) Energy-dispersive spectroscopy (EDS) mapping characterization of ZrMOF .


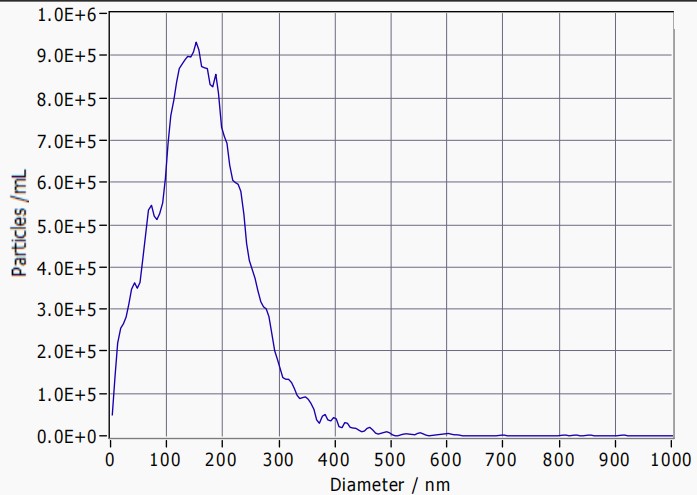


**Figure S3.** Nanoparticle tracking analysis (NTA) of Ru@ZrMOF, showing a median diameter of 156.5 nm.


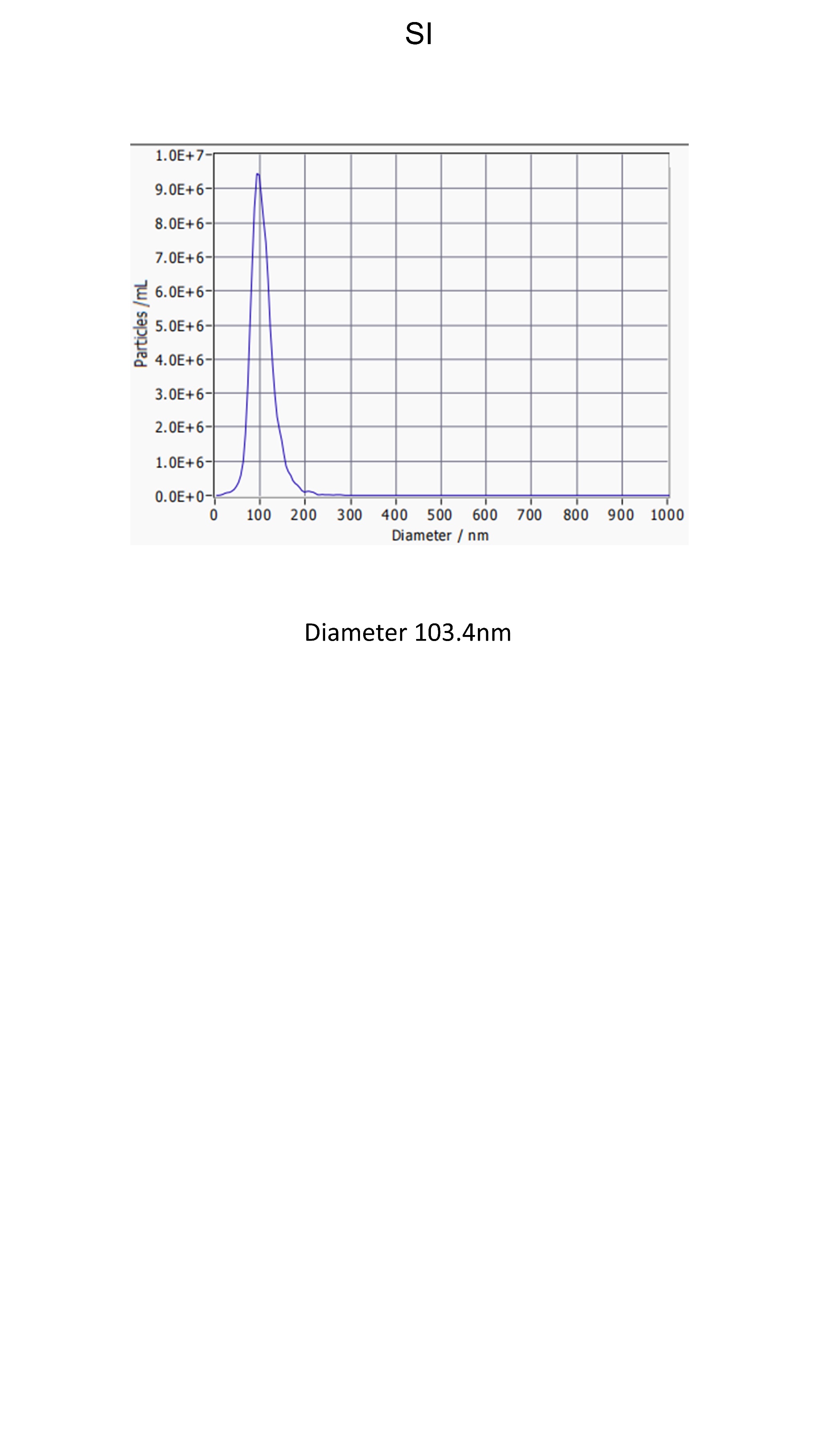


**Figure S4.** NTA result of the size of EVs, showing a median diameter of 99.5 nm.


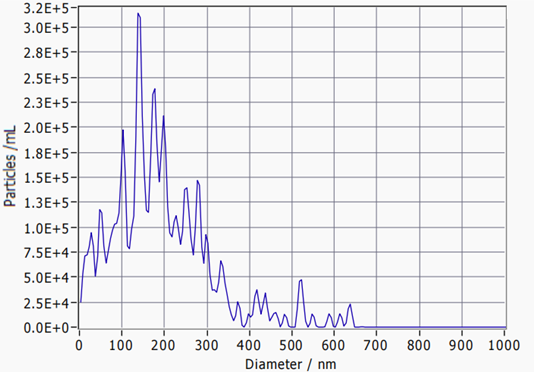


**Figure S5**. NTA result of the size of Ru@ZrMOF/EVs, showing a median diameter of 173.1 nm.


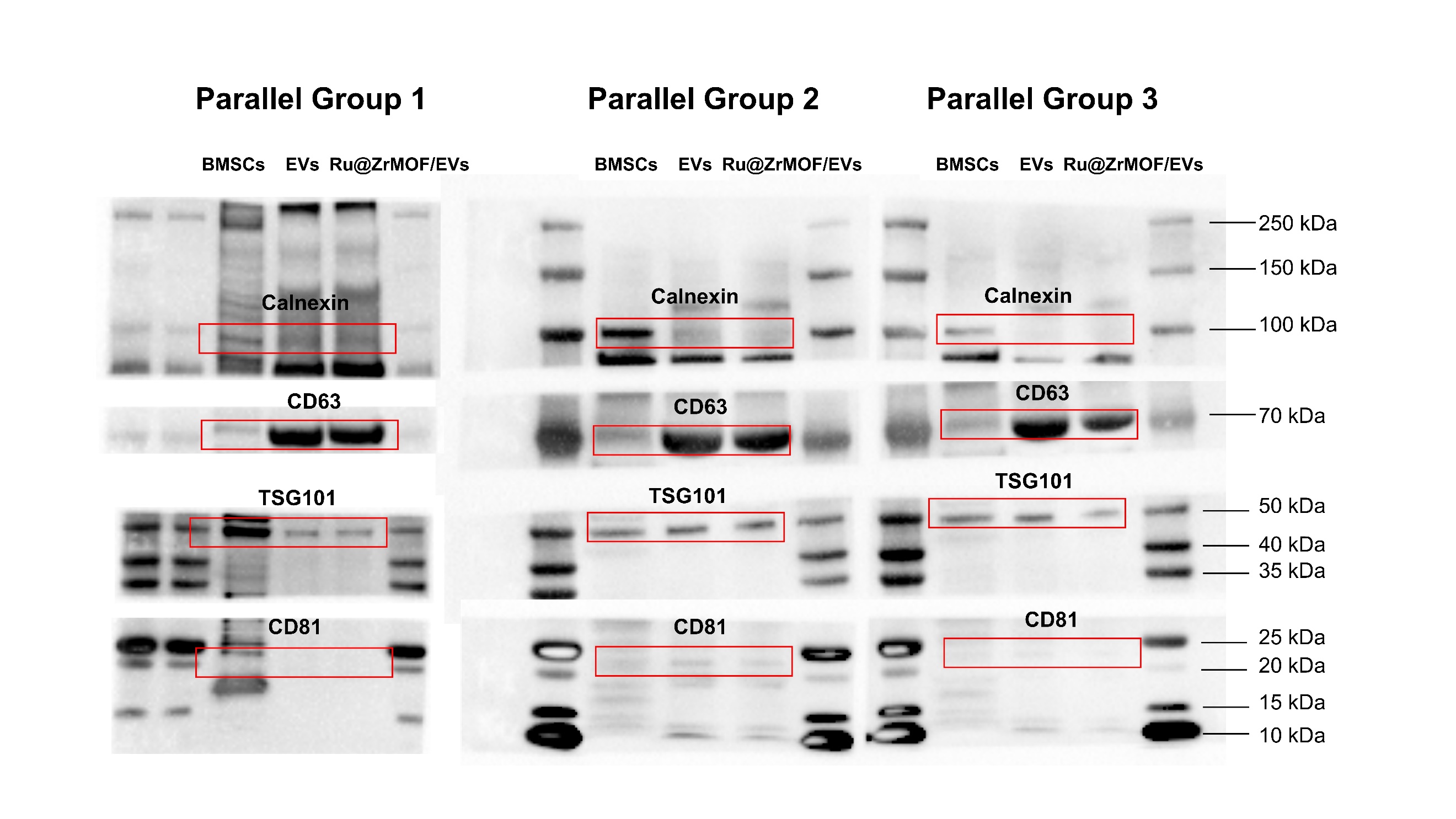


**Figure S6.** Unmodified raw Western blot (WB) images showing the expression of calnexin, CD63, TSG101, and CD81 expressed by BMSCs, EVs and Ru@ZrMOF/EVs. Results are representative blots from n=3 independent experiments.

**
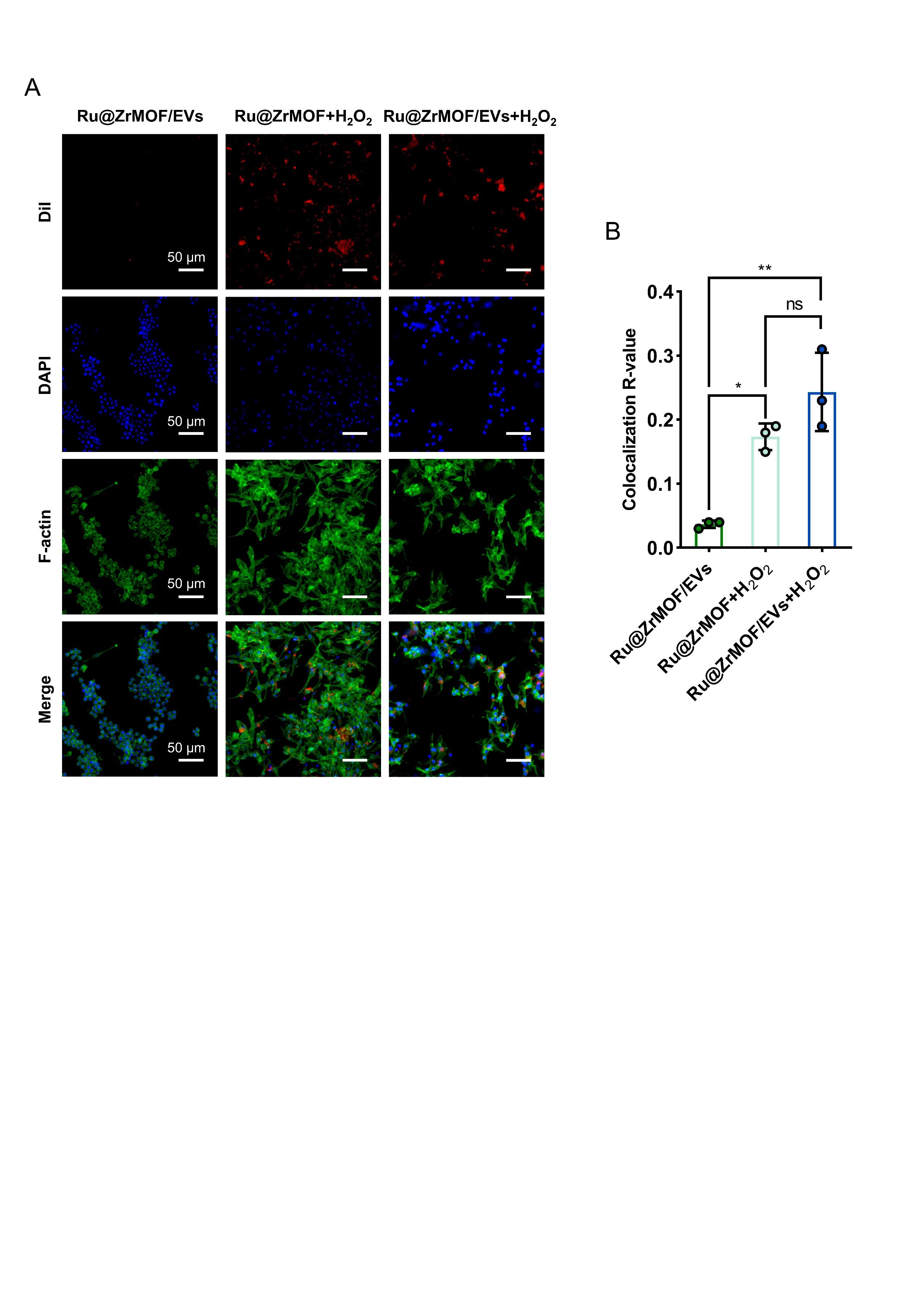
**

**Figure S7.** A) All channels of cellular uptake behavior in CLSM images. B) Statistical analysis of fluorescence colocalization R-values, n = 3.


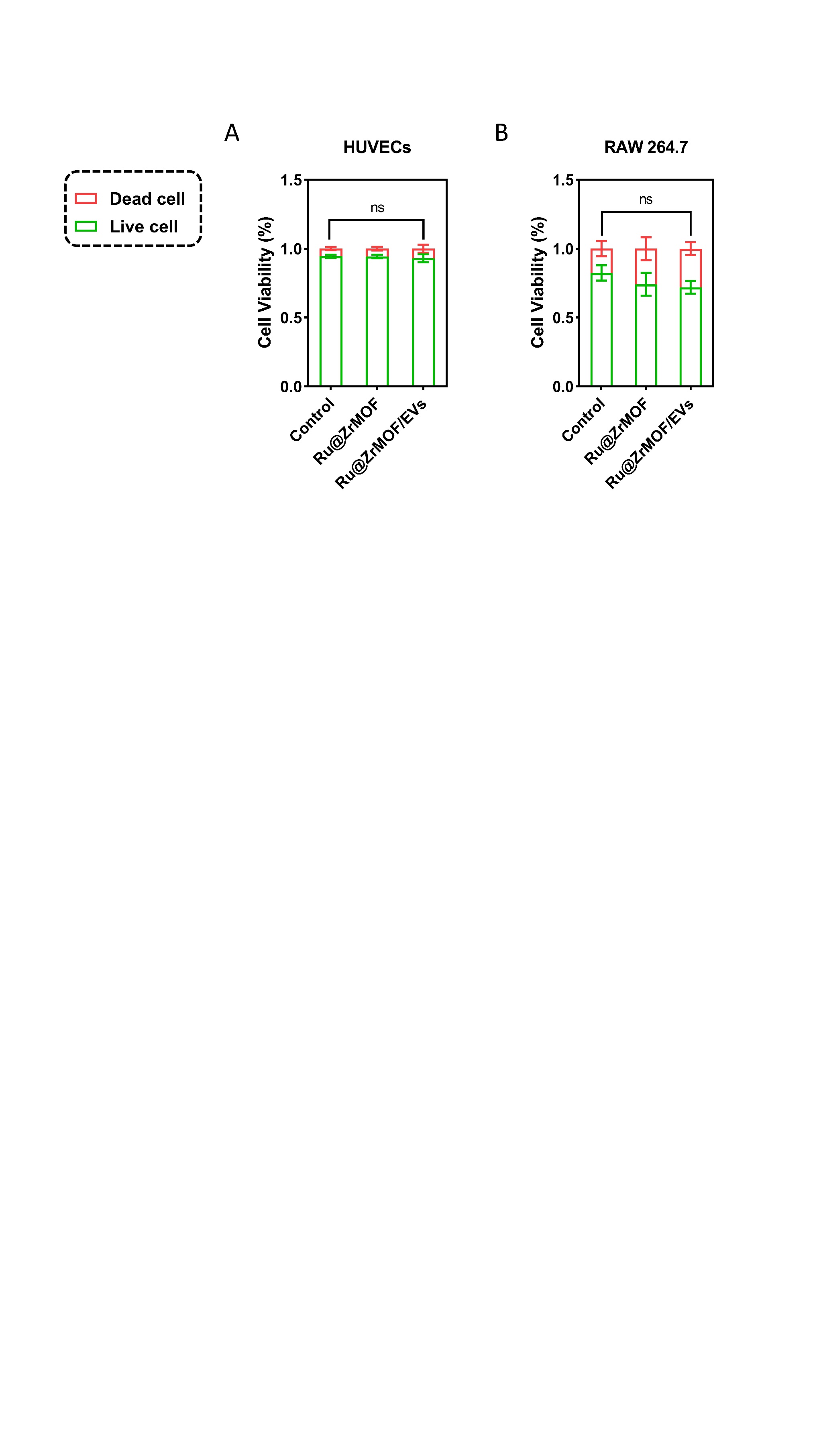


**Figure S8.** Fluorescence images of A) HUVECs and B) RAW 264.7 cells co-cultured with Calcein-AM/PI-stained 4T1 cells after different treatments.


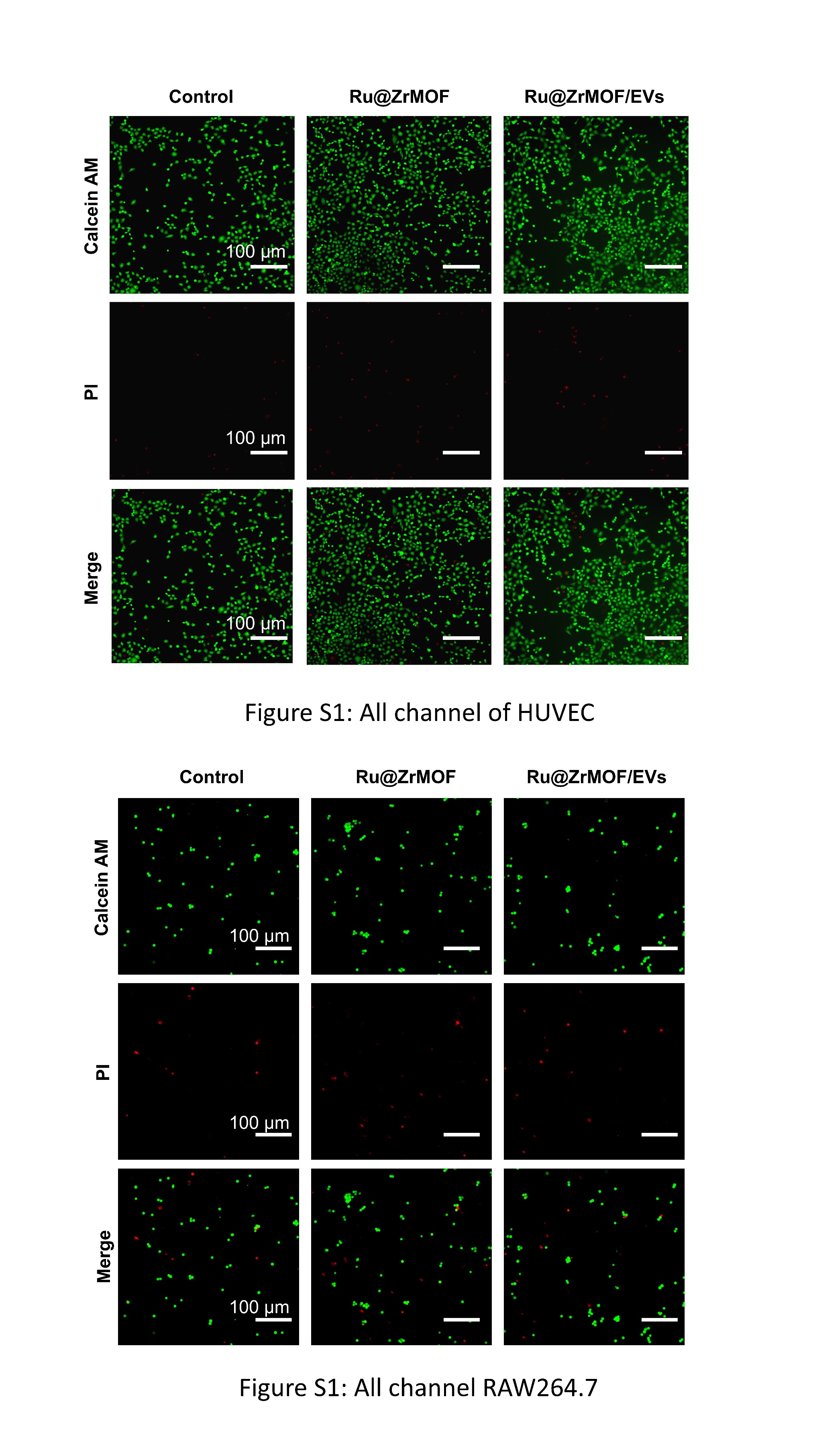


**Figure S9**. All channels of Calcein-AM/PI dual-fluorescence stained HUVECs.


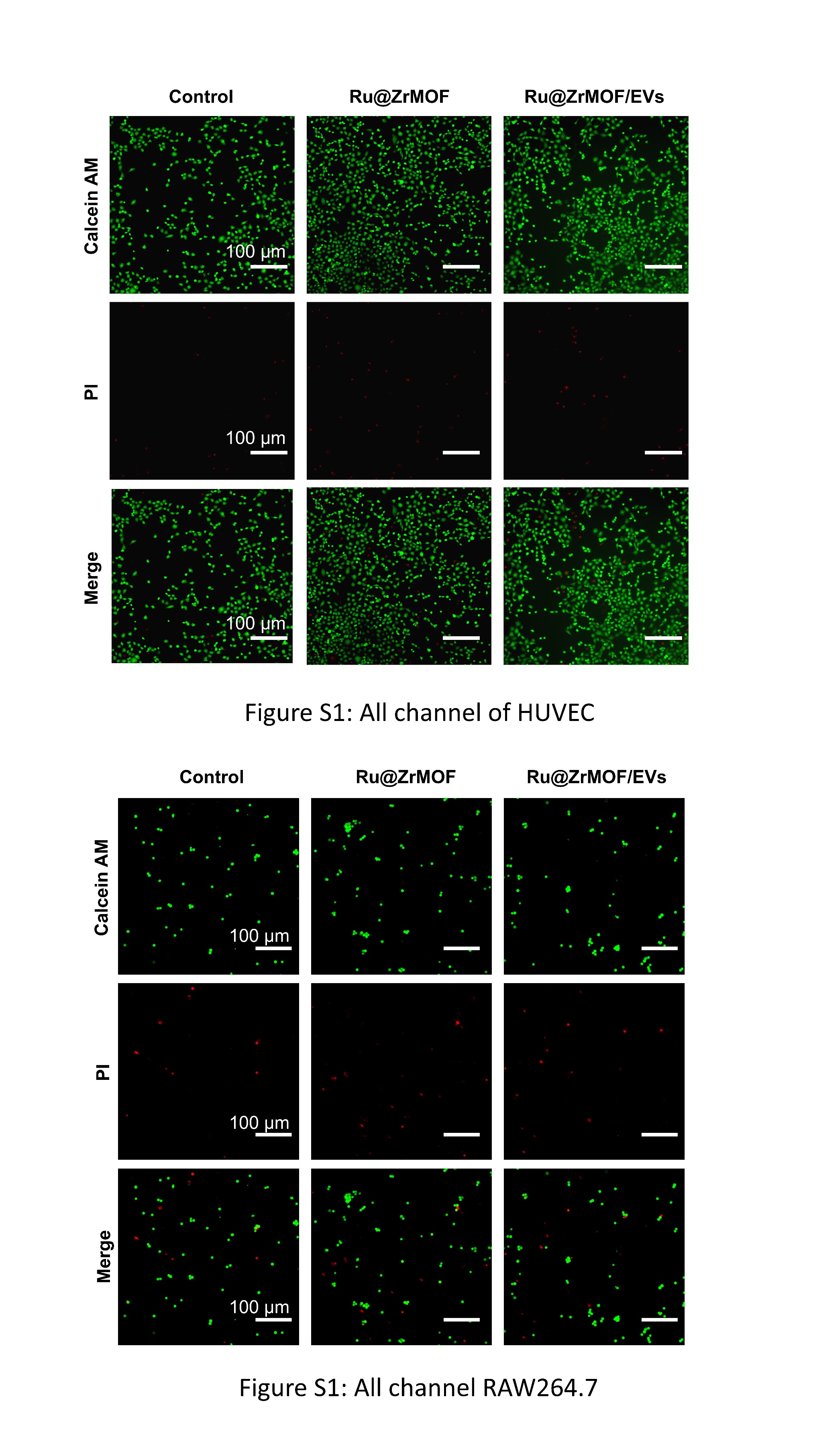


**Figure S10**. All channels of Calcein-AM/PI dual-fluorescence stained RAW 264.7.


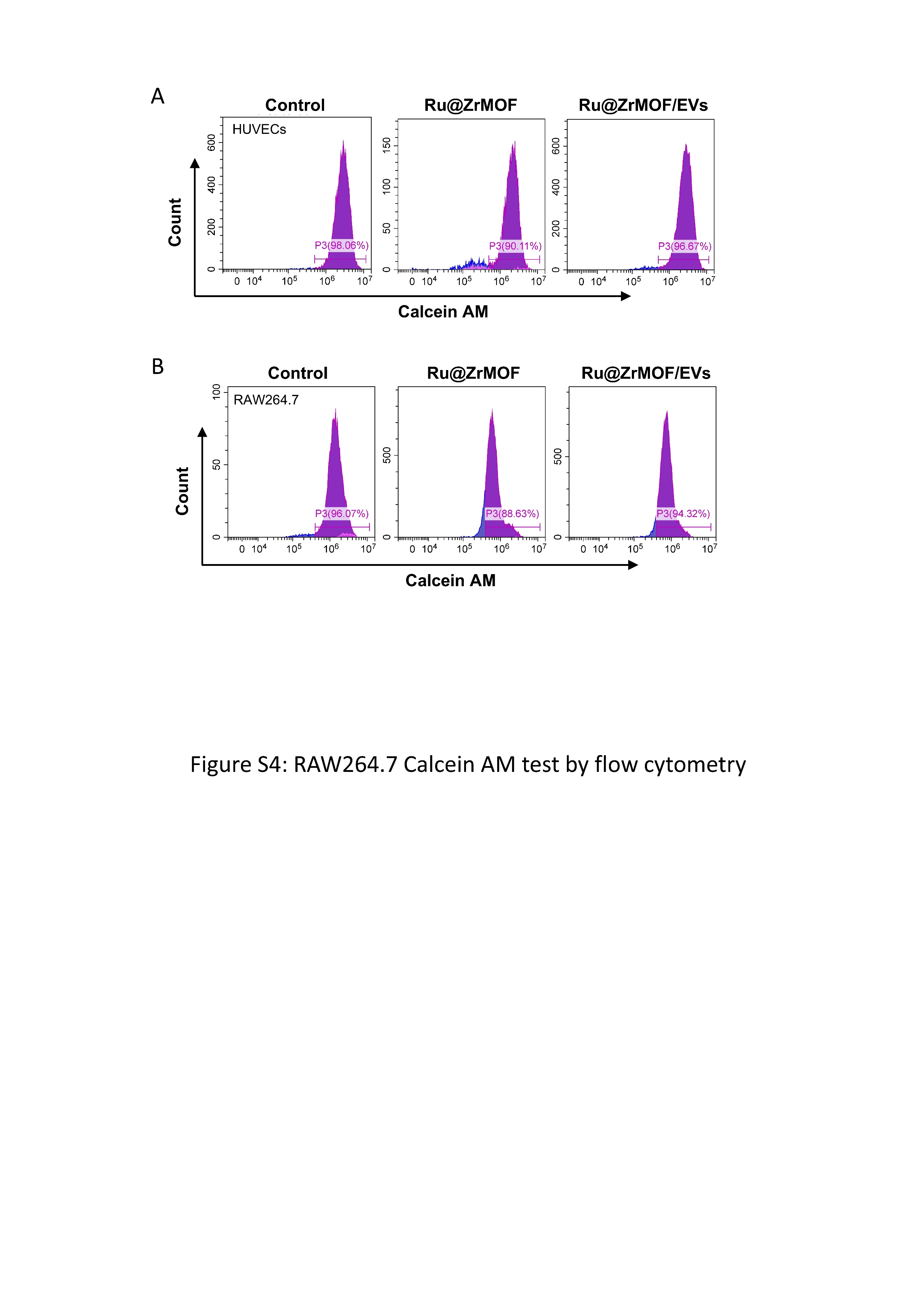


**Figure S11.** A) HUVECs and B) RAW264.7 Calcein AM fluorescence intensity test by flow cytometry.


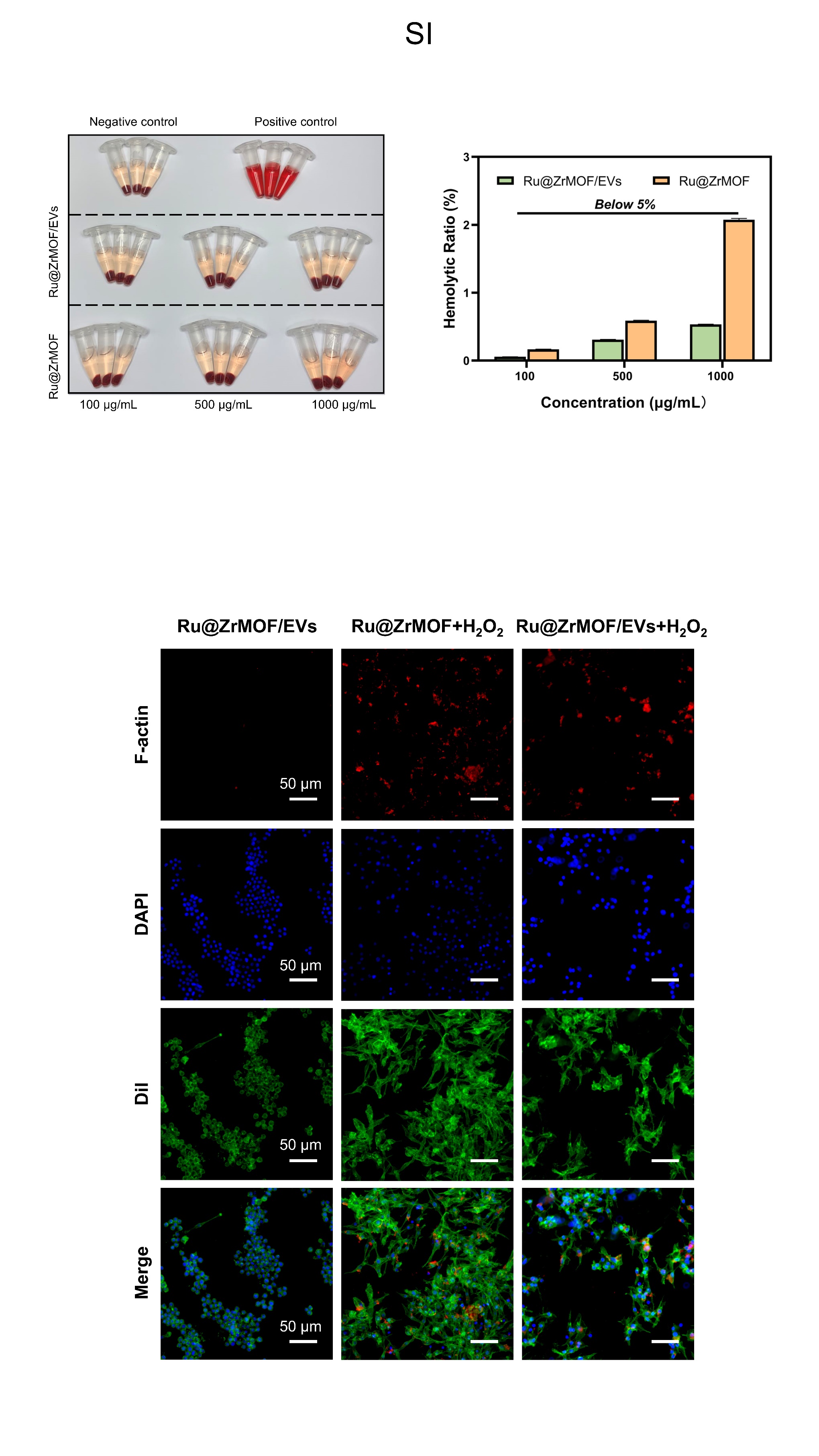


**Figure S12.** Analysis of hemolytic ratio of Ru@ZrMOF and Ru@ZrMOF/EVs at different concentrations.


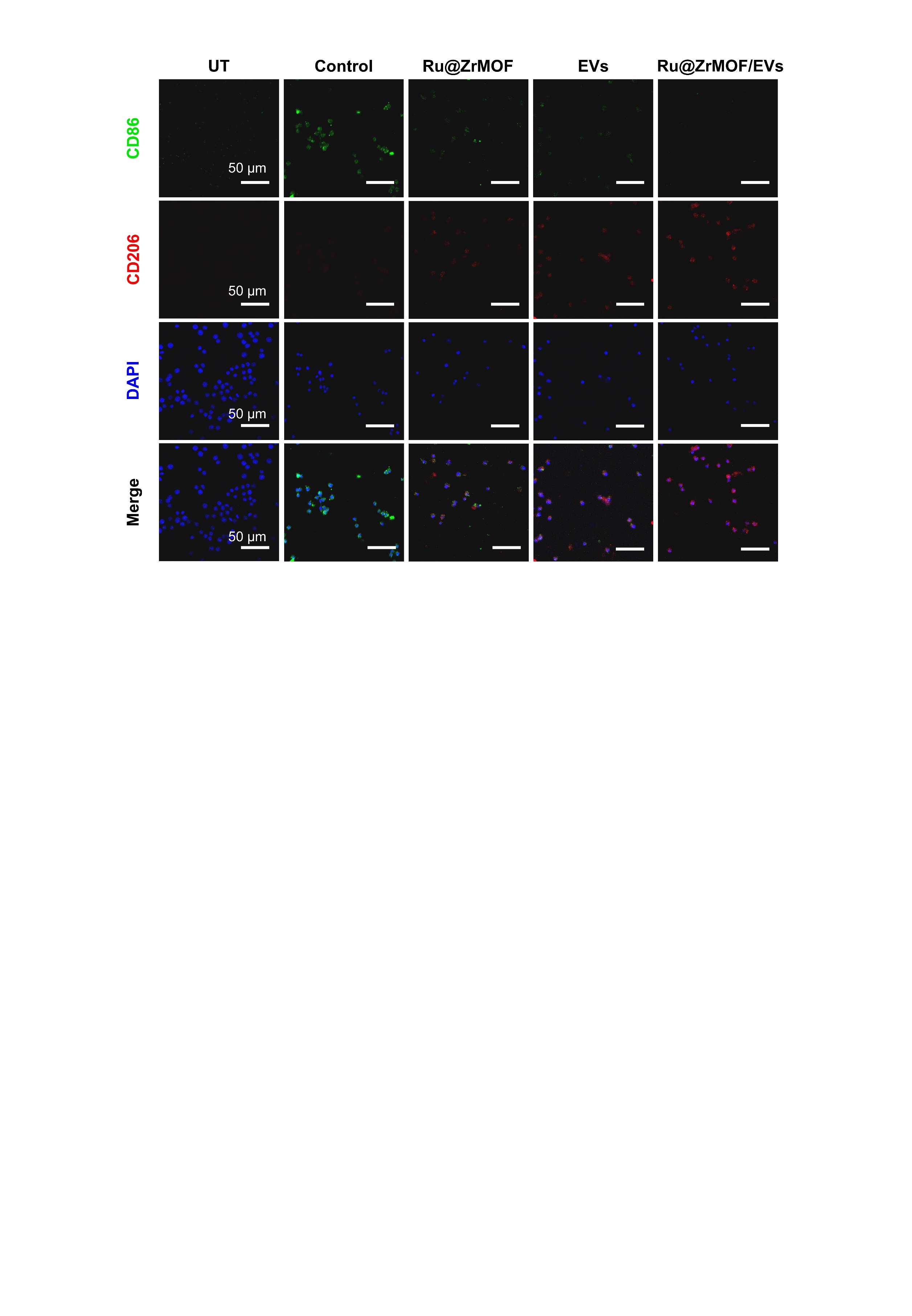


**Figure S13.** All channels of immunofluorescence images of CD86 (green) and CD206 (red) staining of RAW264.7 after Ru@ZrMOF/EVs treatment.


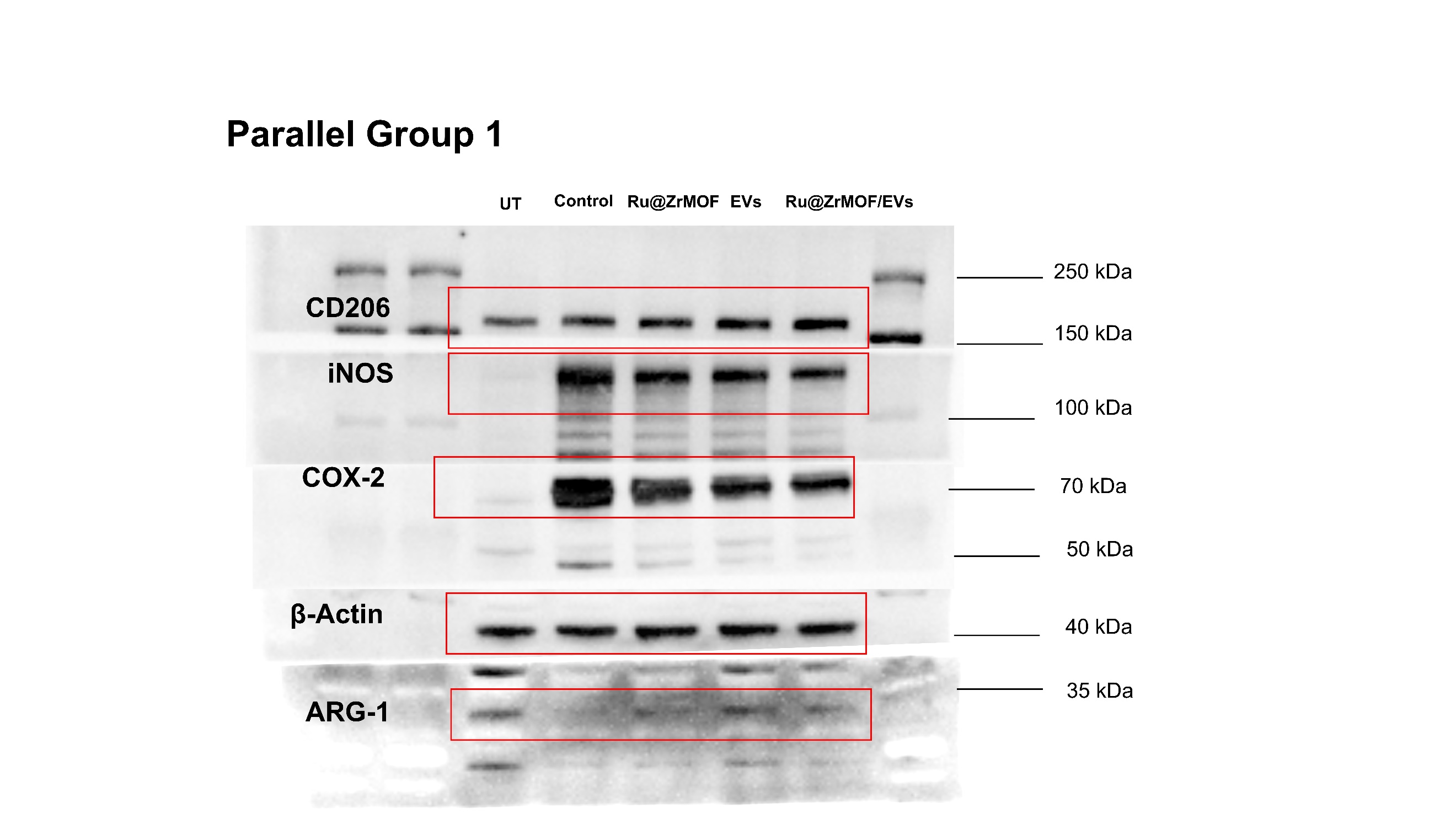


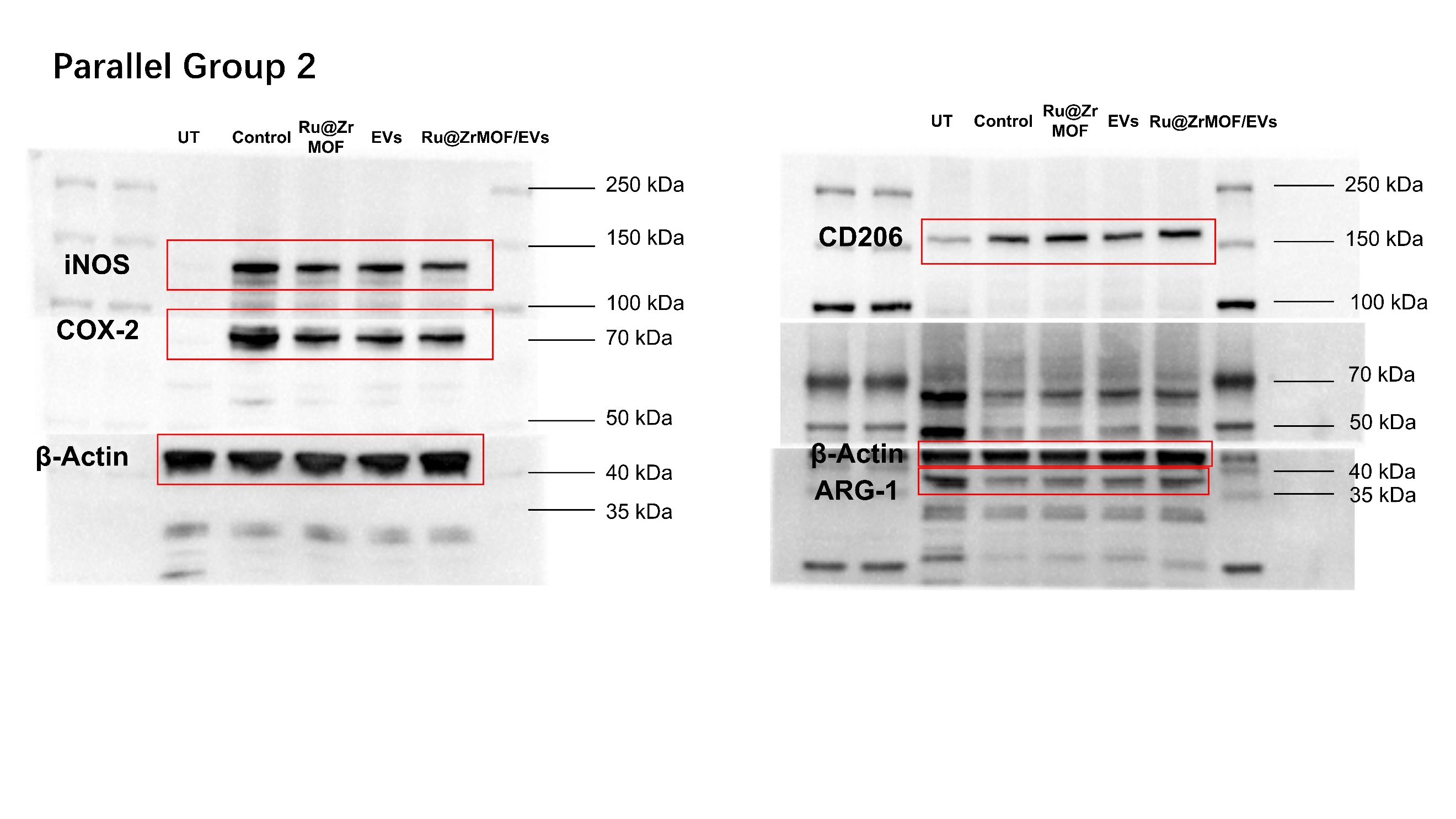


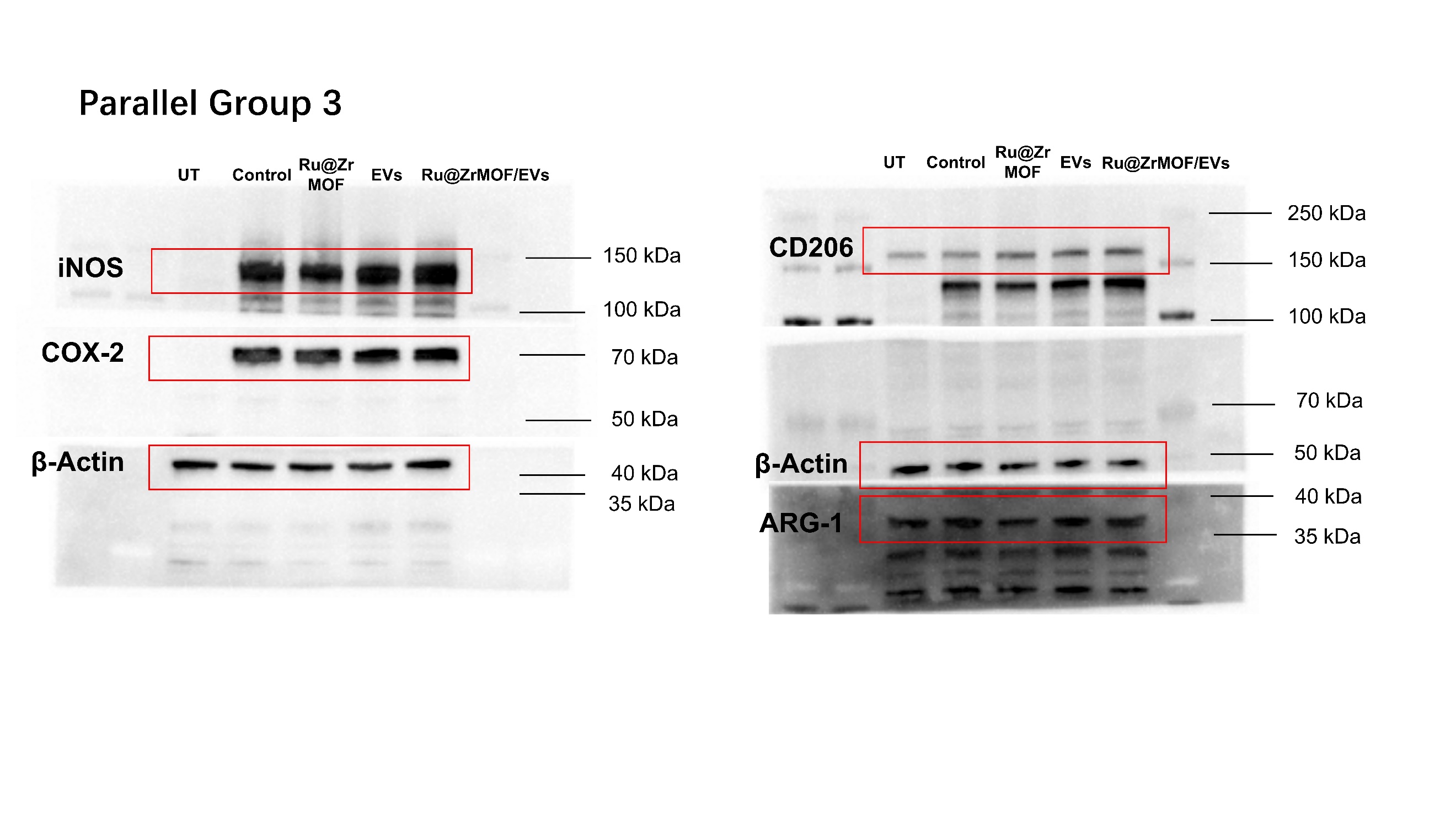


**Figure S14.** Unmodified raw WB image of the M1 markers (iNOS), M2 markers (Arg-1 and CD206) and the inflammatory factor markers (COX-2). Shown are representative blots from n=3 independent experiments.


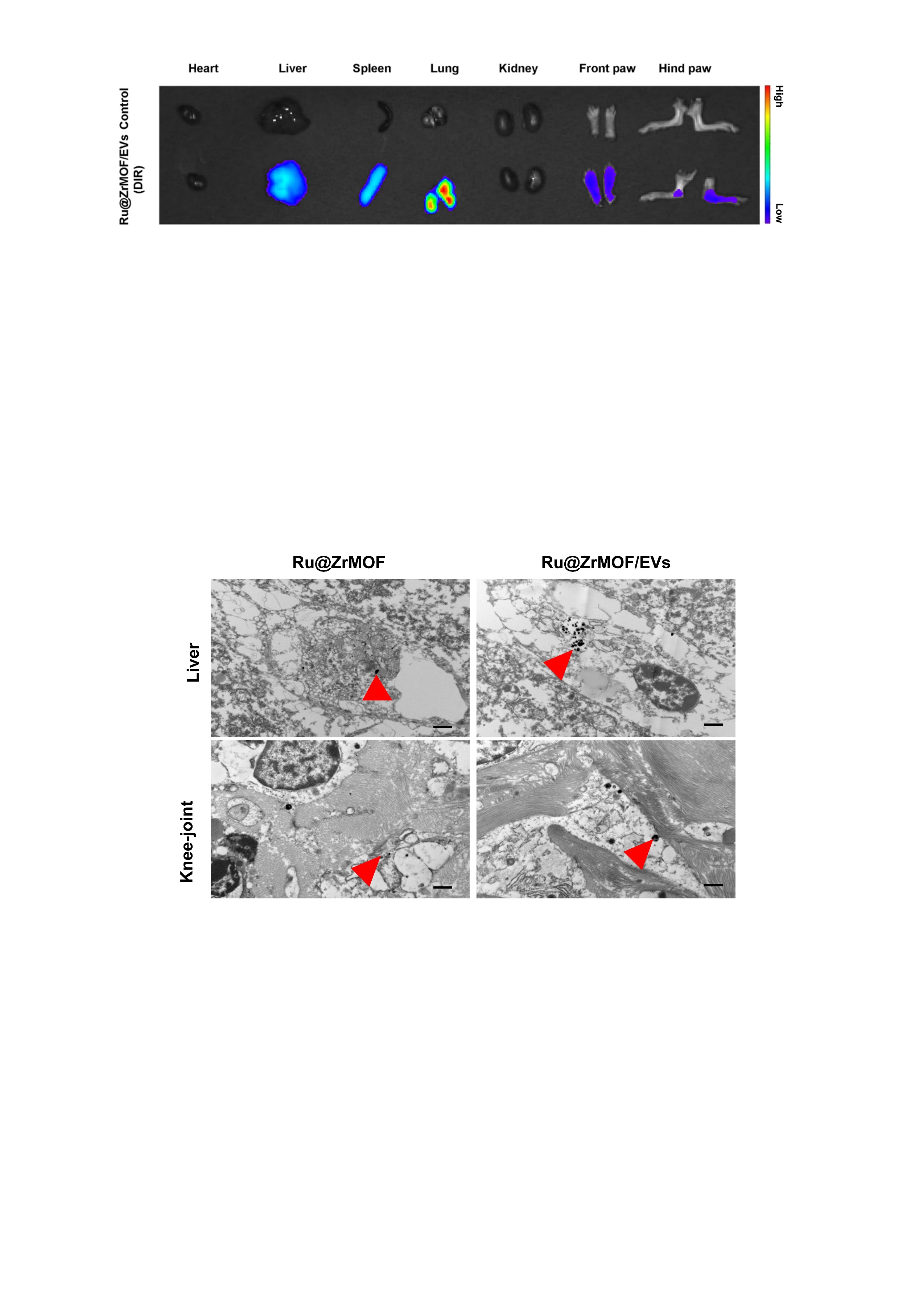


**Figure S15.** The ex vivo fluorescence images of Ru@ZrMOF/EVs in major organs and limbs.


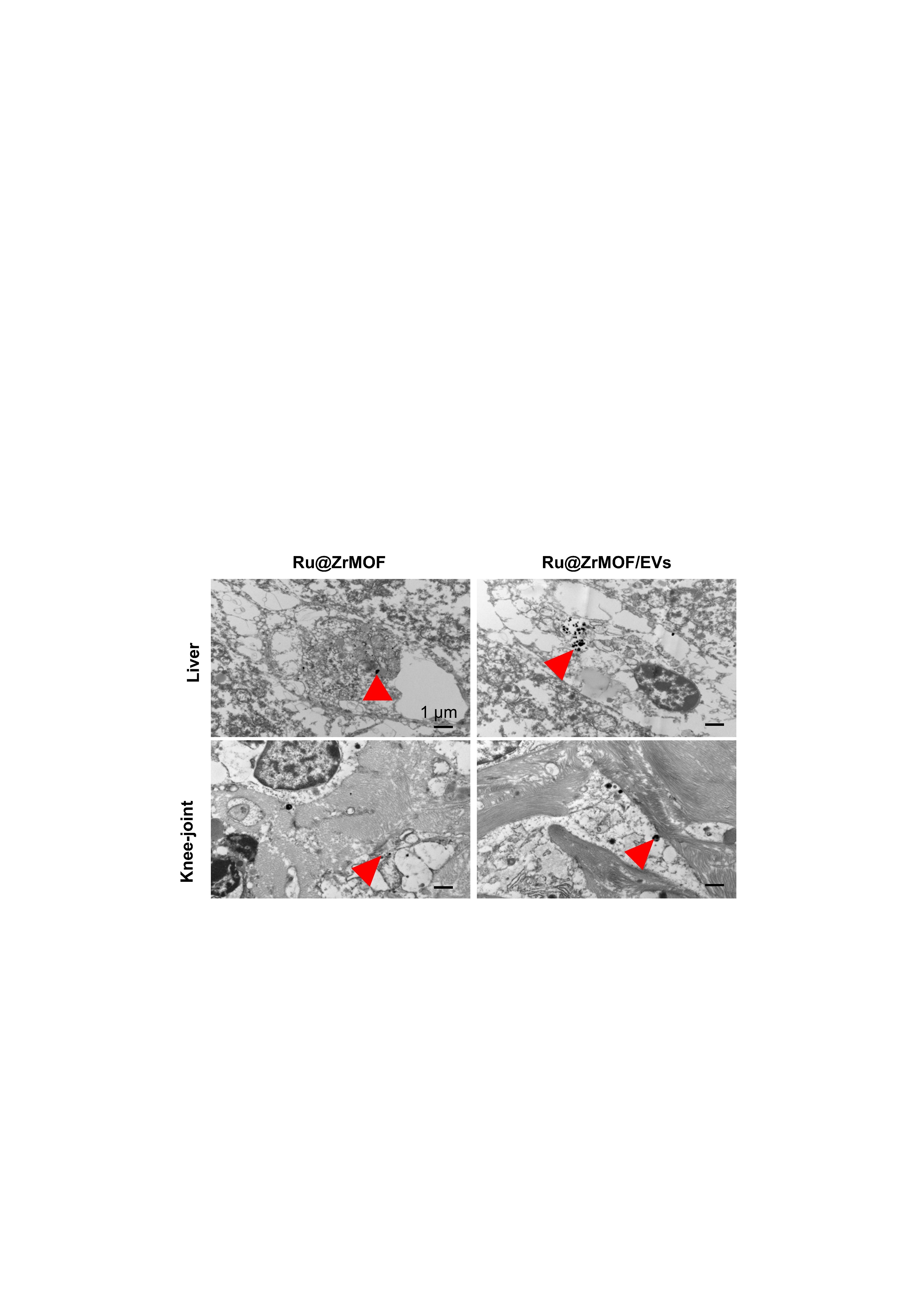


**Figure S16.** TEM images of isolated liver and ankle joints, treated with Ru@ZrMOF and Ru@ZrMOF/EVs (red triangles pointing to indicate nanoparticles).

**
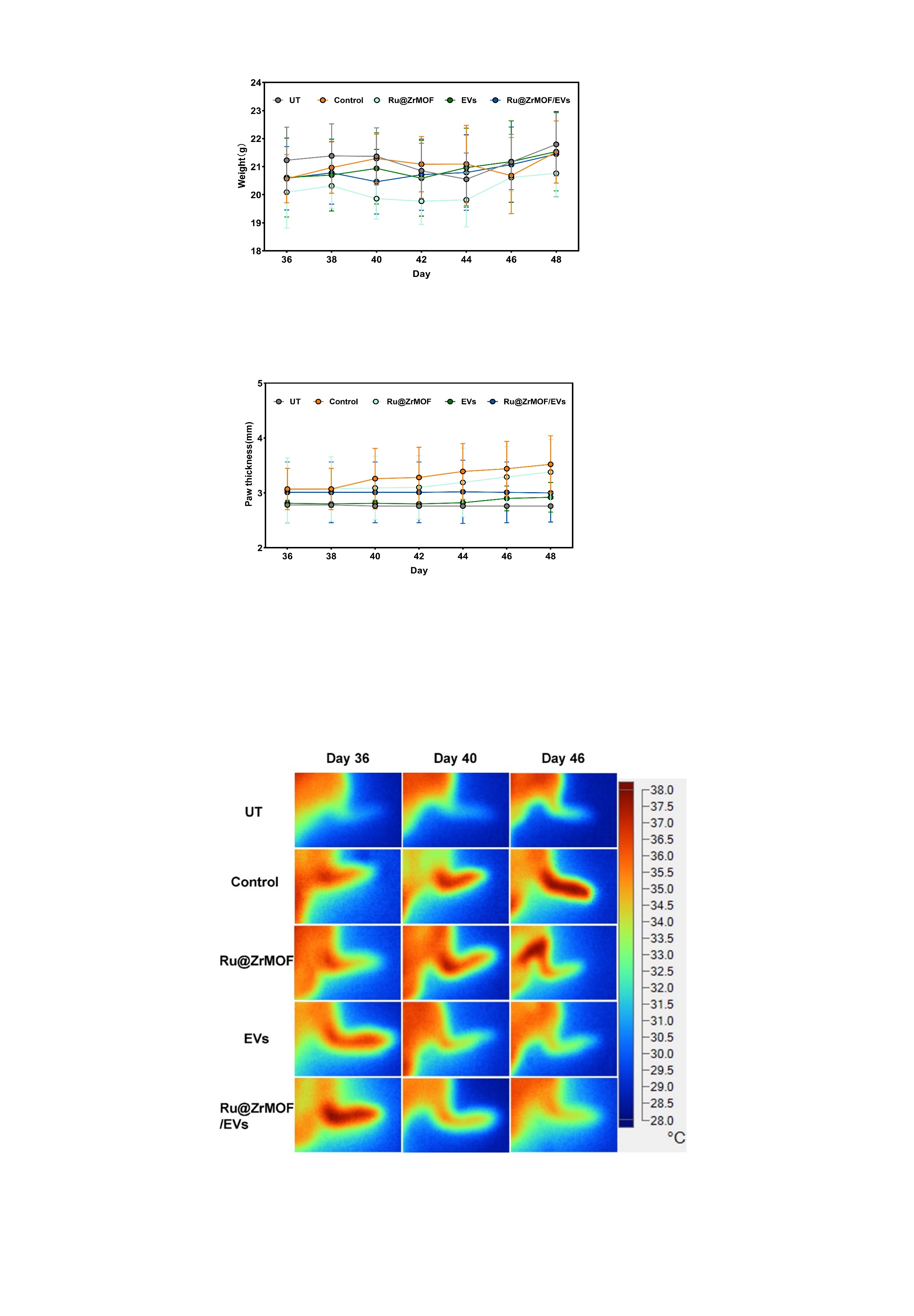
**

**Figure S17.** The hind paw thickness during the treatments, n = 5 per group (five independent experiments).

.


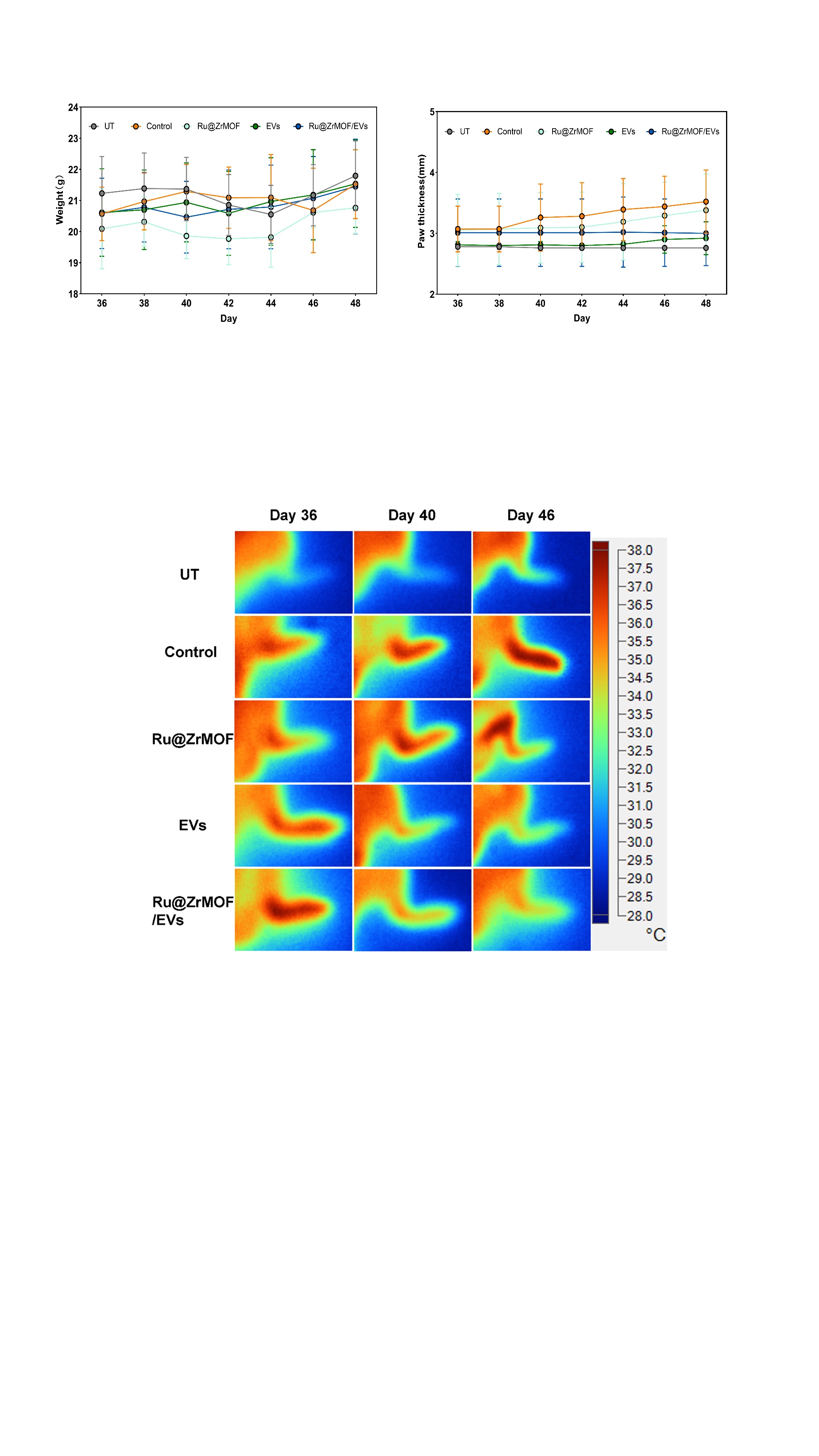


**Figure S18.** Thermal imaging of hindlimb joints at different days in each group of CIA mice

**
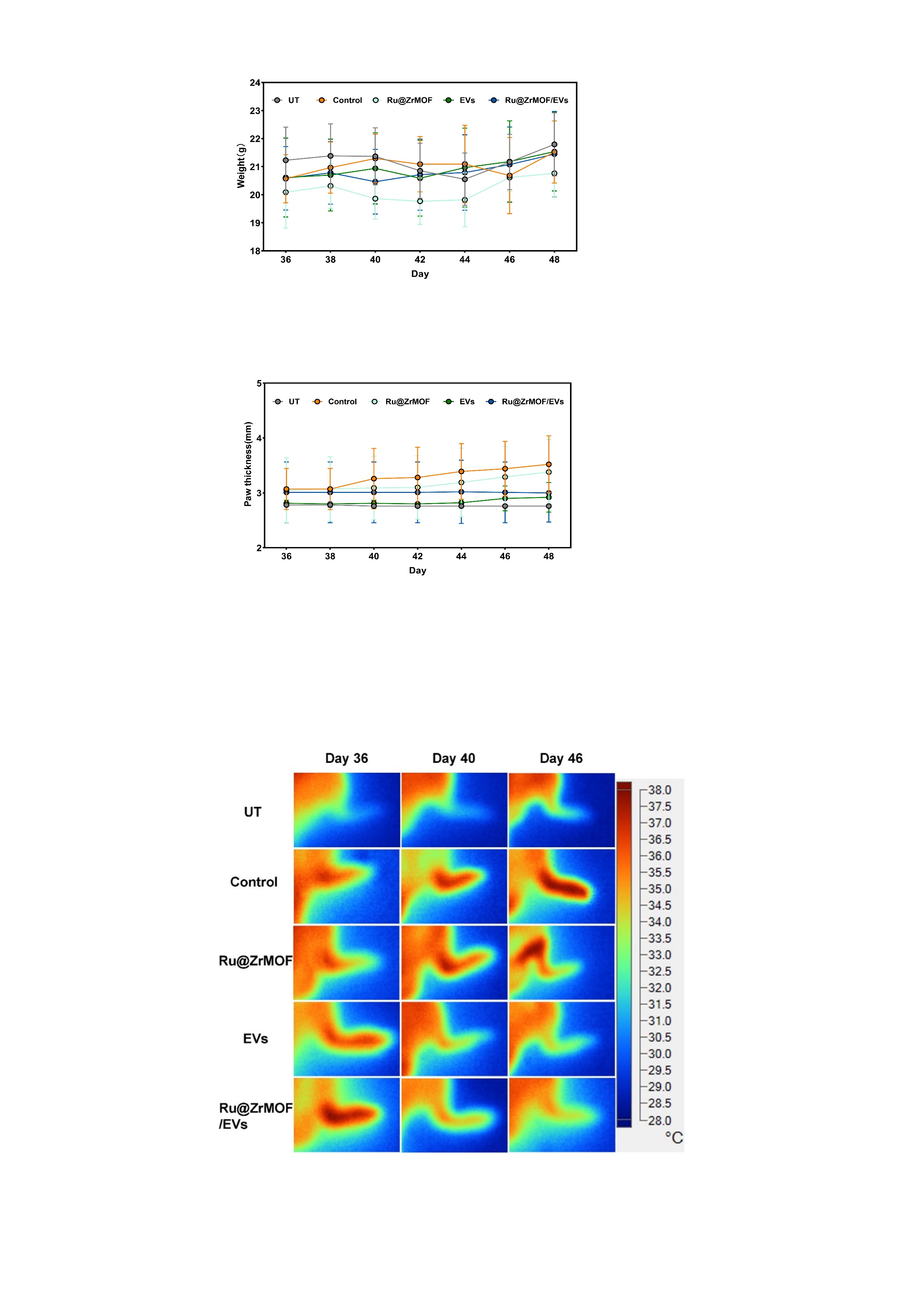
**

**Figure S19.** Weight of different groups during the treatments, n = 5 per group (five independent experiments).


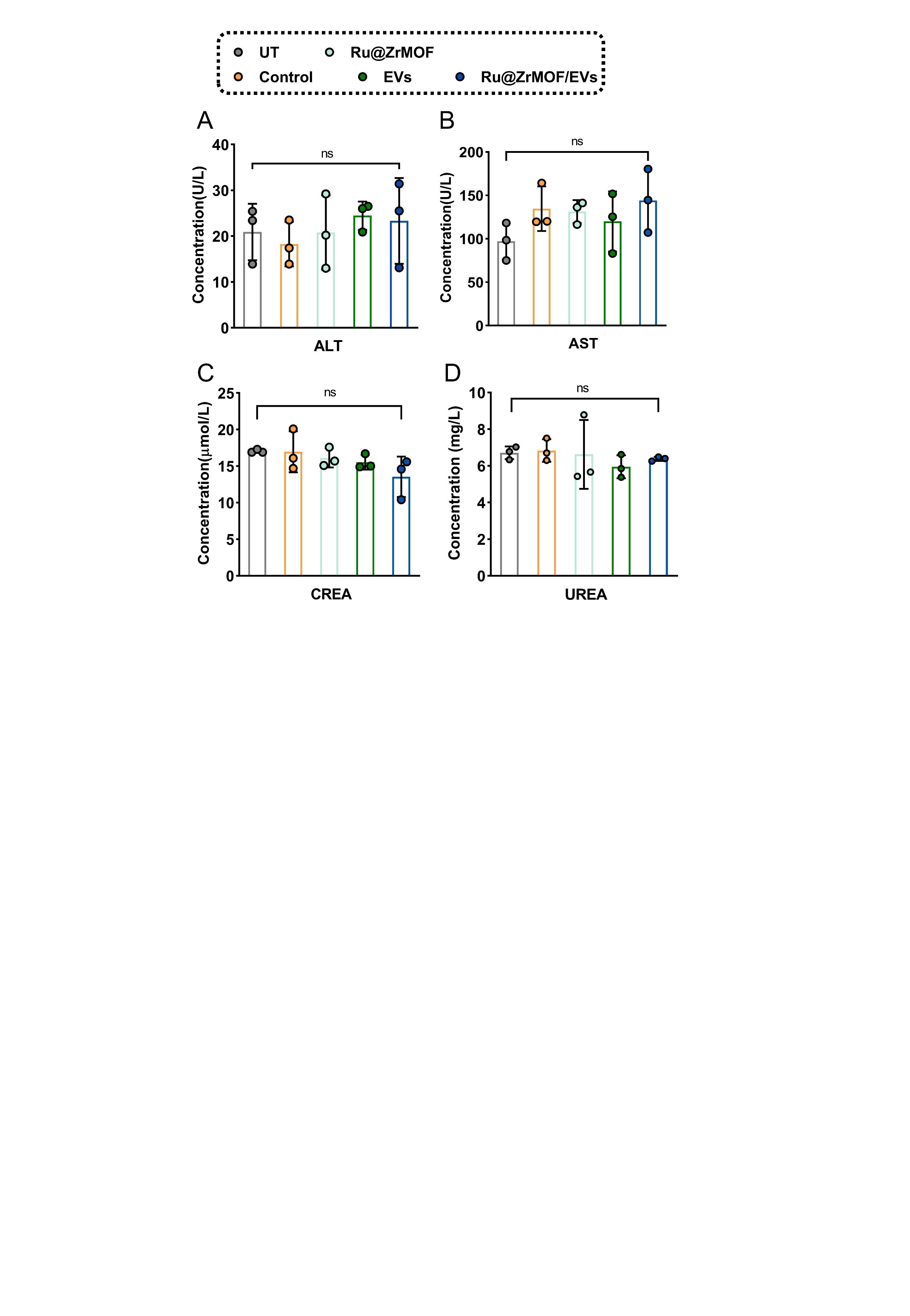


**Figure S20.** Serum biochemistry data including A) alanine aminotransferase (ALT), B) aspartate aminotransferase (AST), C) creatinine (CREA), D) blood urea nitrogen (UREA) was measured, n = 3 per group (three independent experiments). Results are presented as means ± SD, ns represents no significant difference; statistical significance was calculated using one-way ANOVA followed by Tukey’s post-hoc test for multiple comparisons, all tests were two-sided.


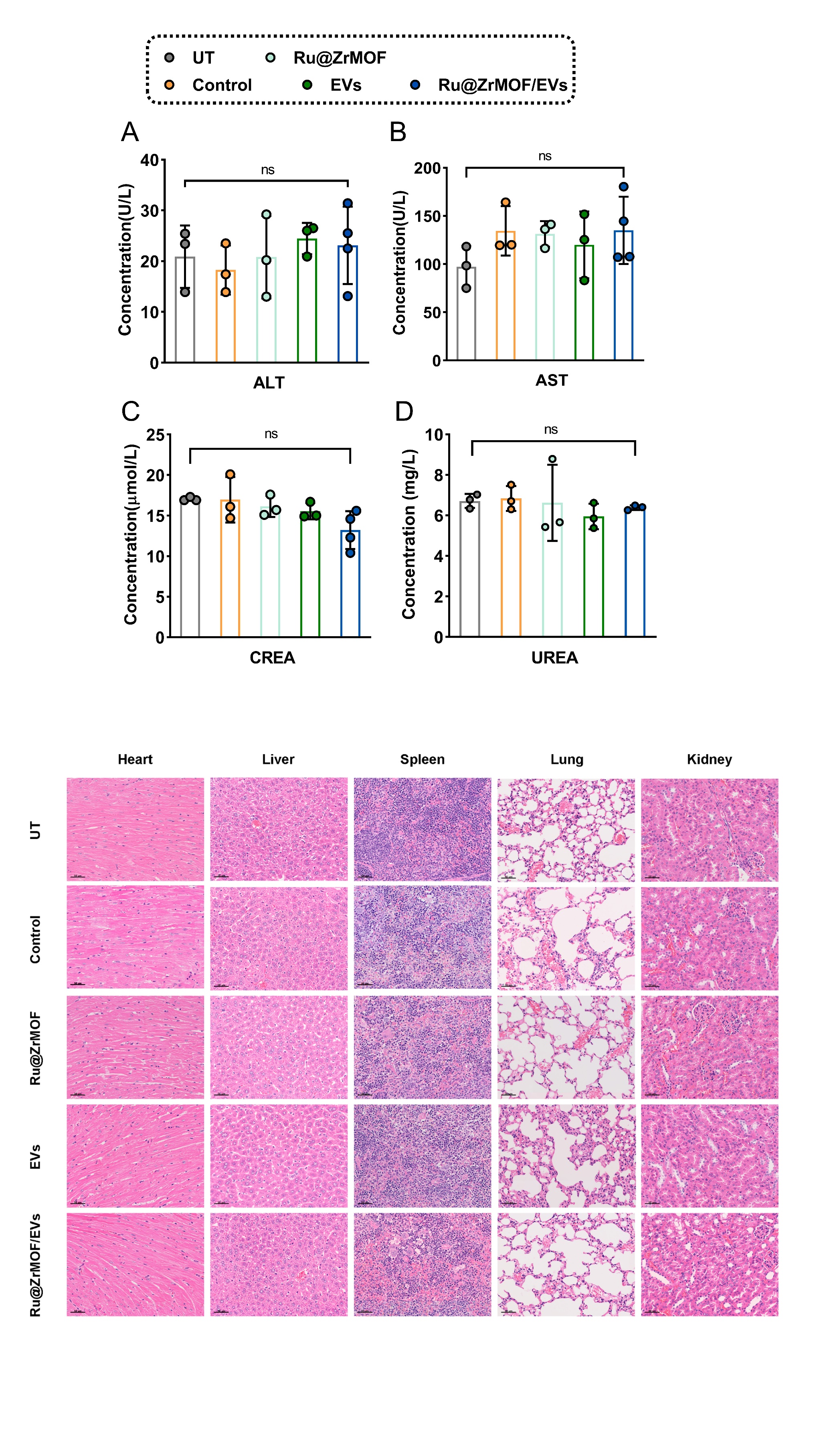


**Figure S21.** H&E-stained tissue sections of major organs (heart, liver, spleen, lung, and kidney) from mice with different treatments, all scale bar = 50 μm.


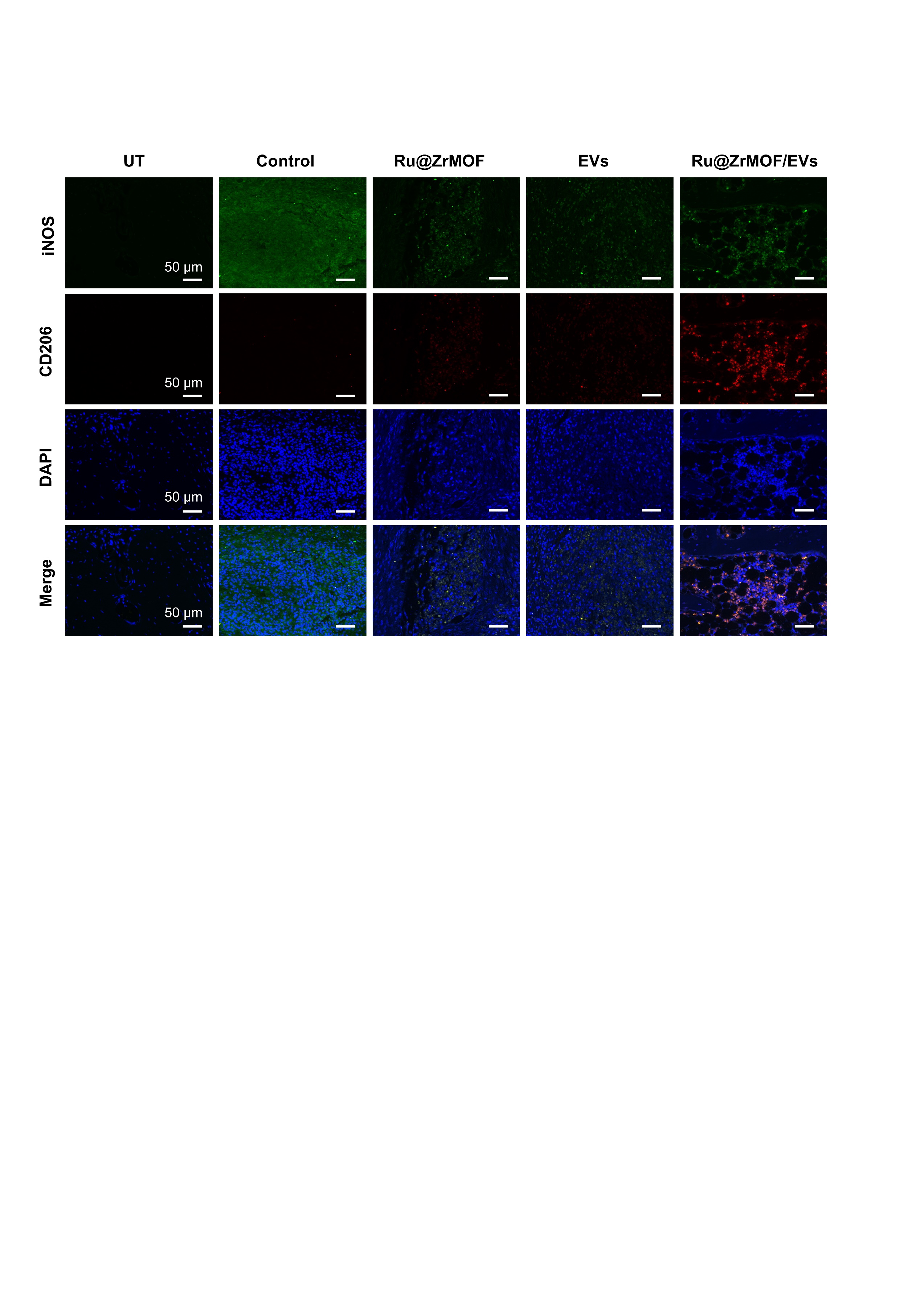


**Figure S22.** All channels of immunofluorescent images of iNOS (green) and CD206 (red) positive cells.


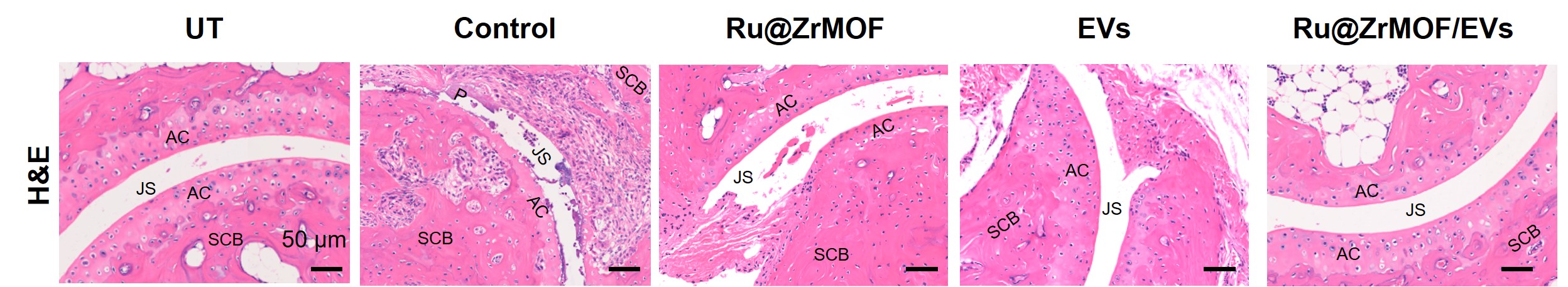


**Figure S23.** Representative H&E-stained images of all experimental groups. AS: articular cartilage; SCB: subchondral bone; JS: joint space; P: pannus.


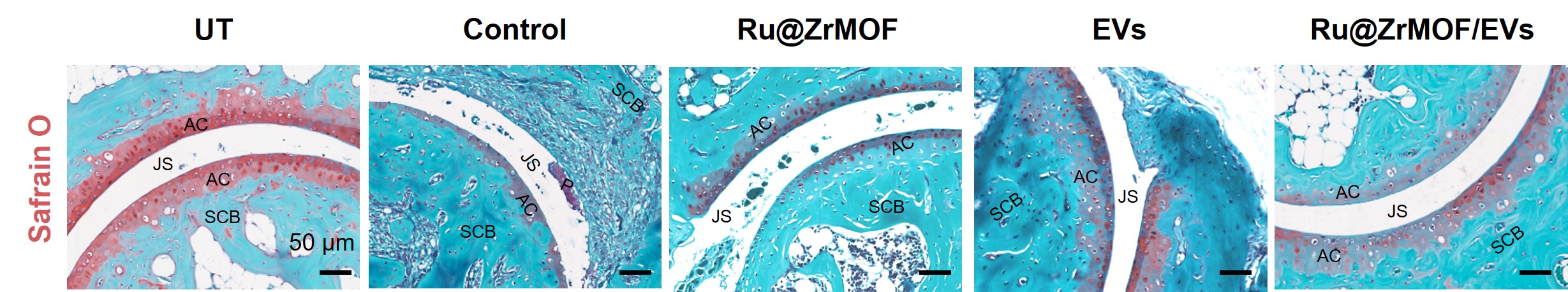


**Figure S24.** Representative Safranin O-fast green staining images of all experimental groups. AS: articular cartilage; SCB: subchondral bone; JS: joint space; P: pannus.


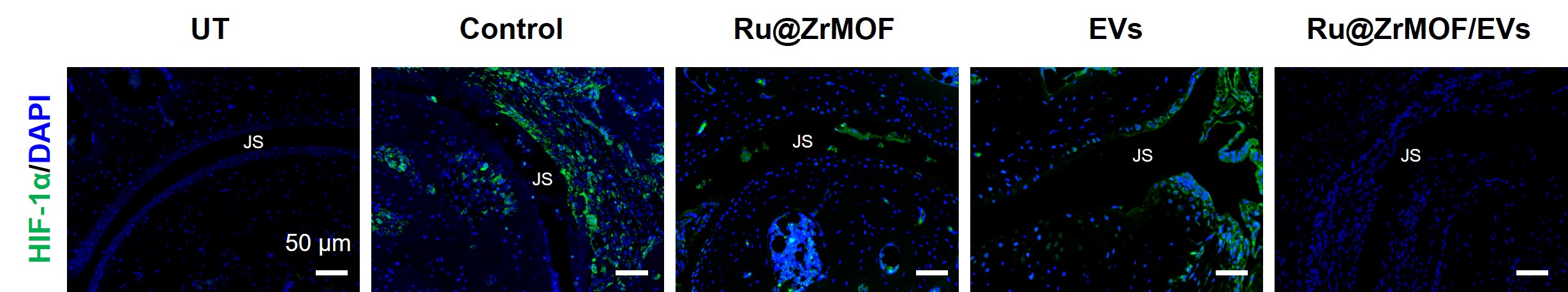


**Figure S25.** Representative immunofluorescent images of all experimental groups of HIF-1α. JS: joint space.


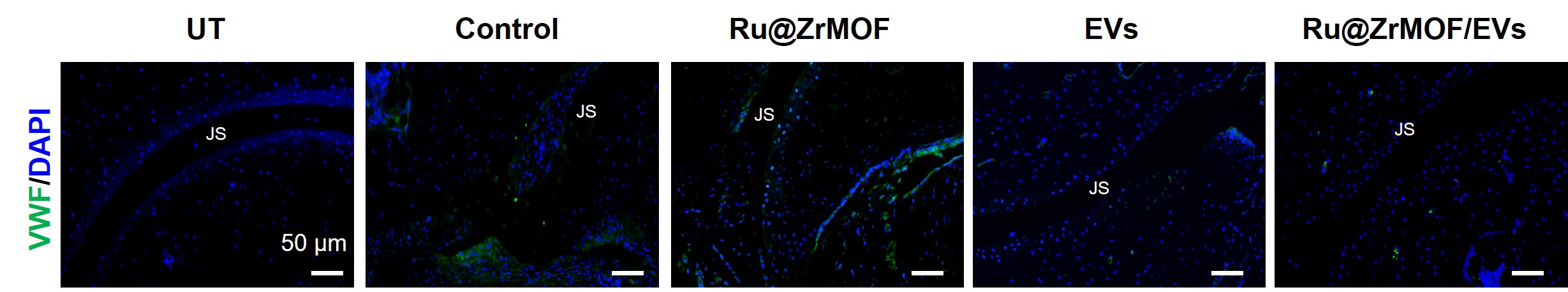


**Figure S26.** Representative immunofluorescent images of all experimental groups of VWF. JS: joint space.

**Table S1**. RT-qPCR primer sequence.

| Target gene | Primer | Sequence |
| --- | --- | --- |
| β-actin | Forward | GCTGCGCTGGTCGTCG |
| β-actin | Reverse | GGCCTCGTCACCCACATAG |
| iNOS | Forward | ACCATGAGGCTGAAATCCCA |
| iNOS | Reverse | TCCACAACTCGCTCCAAGAT |
| IL-10 | Forward | GCTCCAAGACCAAGGTGTCT |
| IL-10 | Reverse | CGGAGAGAGGTACAAACGAGG |
| CD86 | Forward | ACGTATTGGAAGGAGATTACAGCT |
| CD86 | Reverse | TCTGTCAGCGTTACTATCCCGC |
| CD206 | Forward | ACGAGCAGGTGCAGTTTACA |
| CD206 | Reverse | ACATCCCATAAGCCACCTGC |
| IL-1β | Forward | CTGTGACTCATGGGATGATGATG |
| IL-1β | Reverse | CGGAGCCTGTAGTGCAGTTG |
| TNF-α | Forward | TGAGGTCAATCTGCCCAAGT |
| TNF-α | Reverse | GGGGTCAGAGTAAAGGGGTC |
| Arg-1 | Forward | GTAGACAAGCTGGGGATTGG |
| Arg-1 | Reverse | TCAAAGCTCAGGTGAATCGG |

**REFERENCES**

[1] X. Rong, Y. Tang, S. Cao, S. Xiao, H. Wang, B. Zhu, S. Huang, M. Adeli, R. D. Rodriguez, C. Cheng, L. Ma, L. Qiu, *ACS Nano* **2023**, 17, 16501.
